# Mitogenomic Characterization and Phylogenetic Placement of African Hind, *Cephalopholis taeniops*: Shedding Light on the Evolution of Groupers (Serranidae: Epinephelinae)

**DOI:** 10.3390/ijms25031822

**Published:** 2024-02-02

**Authors:** Shantanu Kundu, Hye-Eun Kang, Ah Ran Kim, Soo Rin Lee, Eun-Bi Kim, Muhammad Hilman Fu’adil Amin, Sapto Andriyono, Hyun-Woo Kim, Kyoungmi Kang

**Affiliations:** 1 Institute of Fisheries Science, Pukyong National University, Busan 48513, Republic of Korea; shantanu1984@gmail.com; 2 Institute of Marine Life Science, Pukyong National University, Busan 48513, Republic of Korea; kanghe24@pukyong.ac.kr; 3 Marine Integrated Biomedical Technology Center, National Key Research Institutes in Universities, Pukyong National University, Busan 48513, Republic of Korea; ahrankim@pukyong.ac.kr (A.R.K.); srlee090@pukyong.ac.kr (S.R.L.);; 4 Ocean Georesources Research Department, Korea Institute of Ocean Science and Technology, Busan 49111, Republic of Korea; kimeb@kiost.ac.kr; 5 Advance Tropical Biodiversity, Genomics, and Conservation Research Group, Department of Biology, Faculty of Science and Technology, Airlangga University, Surabaya 60115, Indonesia; m-hilman-f-a@fst.unair.ac.id; 6 Department of Marine, Faculty of Fisheries and Marine, Airlangga University, Surabaya 60115, Indonesia; 7 Department of Marine Biology, Pukyong National University, Busan 48513, Republic of Korea; 8 International Graduate Program of Fisheries Science, Pukyong National University, Busan 48513, Republic of Korea

**Keywords:** serranids, Africa, next-generation sequencing, mitogenome, phylogeny, evolution

## Abstract

The global exploration of evolutionary trends in groupers, based on mitogenomes, is currently underway. This research extensively investigates the structure of and variations in *Cephalopholis* species mitogenomes, along with their phylogenetic relationships, focusing specifically on *Cephalopholis taeniops* from the Eastern Atlantic Ocean. The generated mitogenome spans 16,572 base pairs and exhibits a gene order analogous to that of the ancestral teleost’s, featuring 13 protein-coding genes (PCGs), two ribosomal RNA genes (rRNAs), 22 transfer RNA genes (tRNAs), and an AT-rich control region. The mitogenome of *C. taeniops* displays an AT bias (54.99%), aligning with related species. The majority of PCGs in the mitogenome initiate with the start codon ATG, with the exceptions being *COI* (GTG) and *atp6* (TTG). The relative synonymous codon usage analysis revealed the maximum abundance of leucine, proline, serine, and threonine. The nonsynonymous/synonymous ratios were <1, which indicates a strong negative selection among all PCGs of the *Cephalopholis* species. In *C. taeniops*, the prevalent transfer RNAs display conventional cloverleaf secondary structures, except for tRNA-serine (GCT), which lacks a dihydrouracil (DHU) stem. A comparative examination of conserved domains and sequence blocks across various *Cephalopholis* species indicates noteworthy variations in length and nucleotide diversity. Maximum likelihood, neighbor-joining, and Bayesian phylogenetic analyses, employing the concatenated PCGs and a combination of PCGs + rRNAs, distinctly separate all *Cephalopholis* species, including *C. taeniops*. Overall, these findings deepen our understanding of evolutionary relationships among serranid groupers, emphasizing the significance of structural considerations in mitogenomic analyses.

## 1. Introduction

The mitochondrial genome consists of compact, circular molecules, spanning 16–17 kilobase pairs in size, housing highly conserved encoded genes that play an essential role in the vitality of nearly all eukaryotes [1]. The mitogenome is typically composed of 13 protein-coding genes (PCGs), two ribosomal RNAs (rRNAs), 22 transfer RNAs (tRNAs), and two noncoding regions—the control region (CR) and the origin of L-strand replication (OL) [2]. Notably, the gene order within the mitogenome exhibits a remarkable degree of conservation among vertebrates, including teleosts [3]. Due to several advantageous features, such as maternal inheritance and a higher mutation rate, the mitochondrial genome has found extensive use as a powerful tool for conducting phylogenetic and population genetic analyses in vertebrates, with a particular emphasis on fish [4,5]. Consequently, it is imperative to unravel the structure and variability of the mitogenome in any organism to comprehend its functions [6]. While mitogenomic research on fish has been extensively initiated globally, there exists a substantial gap in knowledge within numerous classified groups.

Groupers within the genus *Cephalopholis* (Serranidae: Epinephelinae) represent primarily tropical and subtropical marine fish species, with a global presence encompassing 25 extant species [7,8]. The majority of these species inhabit reef ecosystems extending from the Red Sea to the Indo-Pacific region, while a subset confines their distribution to the Eastern Pacific and the Western to Eastern Atlantic. Economically significant, these species are actively pursued by commercial, artisanal, and recreational fishermen [9,10]. The African hind or blue-spotted seabass, *Cephalopholis taeniops*, inhabits sandy or rocky seafloors at depths ranging from 20 to 200 m in the Eastern Atlantic, spanning from Morocco south to Angola, including the Canary Islands (Spain), Cape Verde Islands, and São Tomé and Principe [7]. Subsequently, this species has been documented in the Canary Islands, the Mediterranean Sea in Libya and Israel, as well as in the Southwestern Atlantic Ocean [10,11,12,13,14,15,16]. Beyond the Eastern Atlantic, populations in the Mediterranean and Southwestern Atlantic are considered introduced populations of *C. taeniops* [16]. Commercially, *C. taeniops* is predominantly utilized for local human consumption within its distribution area and is frequently exported to Europe from Senegal [7,11]. Despite the scarcity of available data regarding population biology, reproductive strategies, and the actual impact of fishing, *C. taeniops* is classified as a ‘Least Concern’ species in the IUCN Red List of Threatened Species due to insufficient evidence. Nevertheless, groupers remain highly vulnerable to fishing pressure, owing to life history traits such as longevity, late sexual maturation, and aggregation spawning [17].

Within the Serranidae family, focused investigations on *Cephalopholis* species have primarily addressed aspects such as physiology and reproductive life history [18,19], genetic connectivity, phylogeography, evolutionary implications through Pleistocene isolation [20,21], and the assembly of chromosome-level genomes alongside transcriptome comparisons [22]. Further, in conjunction with other serranid species, comprehensive examinations of the phylogenetic relationships of *Cephalopholis* species have involved the utilization of two mitochondrial (*16S rRNA* and *12S rRNA*) and two nuclear (*Tmo-4C4* and *histone H3*) genes [23,24]. Numerous molecular datasets have been generated for serranid species, contributing to the discovery of new species [25], the delineation of cryptic diversity [26], species discrimination in hybrid zones [27], and the elucidation of phylogenetic relationships [28,29]. Nevertheless, the systematic classification of serranids has experienced frequent changes across various taxonomic levels, leading to inconsistencies, a situation rectified through the application of three mitochondrial genes (*COI*, *16S rRNA*, and *12S rRNA*) and one nuclear gene (*TMO4C4*) [30]. The resulting molecular data have been instrumental in estimating phylogeographic patterns, demographic history [31,32,33,34], gaining insights into population dynamics and adaptive radiation [35], detecting biological invasions [36], and contributing to the improvement of fisheries management and grouper conservation [37]. Subsequent advancements in molecular tools have further facilitated the exploration of phylogenomics and population genomics within this fish group, unveiling intraspecific variation, adaptation differentiation, physiological changes, radiations, and the intricate processes of speciation [38,39,40].

Examining the complete mitochondrial genome information of serranids, a comprehensive set of 94 species sequences is presently available in the global GenBank database (https://www.ncbi.nlm.nih.gov, accessed on 12 December 2023). Notably, the majority of these mitogenomes have been generated within the subfamily Epinephelinae and the tribe Epinephelini. The database is further enriched by the inclusion of mitogenomes from nine *Cephalopholis* species [41,42,43,44,45,46]. Given the demonstrated efficacy of mitochondrial genomes in elucidating phylogenetic inferences within teleosts [47,48,49], there is an urgent need to enhance taxonomic coverage for a more profound understanding of the evolutionary relationships within the targeted group. Novel mitogenomic data not only provide insights into the structural variations in mitochondrial genes (PCGs, rRNAs, tRNAs, and D-loop), but also shed light on their functions [50,51]. Additionally, mitogenomic data prove valuable in environmental DNA metabarcoding studies and contribute to the conservation genetics of teleosts [52].

Furthermore, the imperative need for the meticulous tracking of tropical marine biodiversity extends to both established hotspots and peripheral ecosystems [53]. The study region, situated within the West African hotspot, recognized as one of the global biodiversity hotspots [54], is characterized by a limited understanding of the diversity, origin, and evolution of reef-associated fish, specifically within *Cephalopholis* species in the Eastern Atlantic Ocean. To overcome these challenges, leveraging genetic information and employing phylogenetic approaches have proven to be successful strategies in elucidating the underlying causes of richness patterns across global marine regions. This involves a comparative assessment of the relative importance of colonization time, the number of colonization events, and diversification rates [55]. The adoption of such integrated approaches is instrumental not only in enhancing our comprehension of these aspects, but also in formulating effective conservation priorities for global marine biodiversity. These priorities, spanning multiple dimensions, play a pivotal role in the context of marine protected areas (MPAs), supporting our capacity to combat and adapt to climate change [56,57]. Therefore, the present study aims to generate a novel mitogenome of the African Hind, *C. taeniops*, from the Eastern Atlantic Ocean, characterizing its genomic features in comparison with other congeners. The research also conducts cladistic analyses to elucidate the evolutionary relationships of the targeted species with other major lineages of serranids. The genetic data acquired will play a crucial role in validating the population structure of the African Hind and its applications in the field of conservation genetics.

## 2. Results and Discussion

### 2.1. Mitogenome Structure and Organization

**Figure 1:**
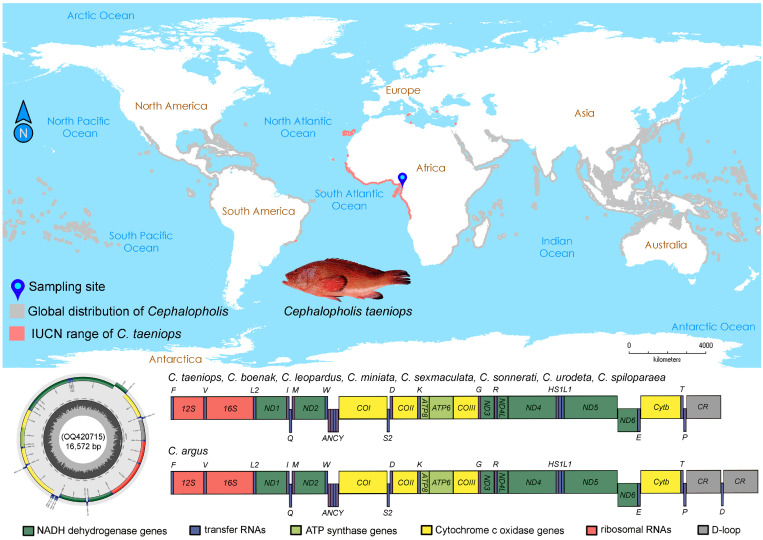
Global distribution pattern of African Hind, *C. taeniops* and other congeners in marine environment. Collection locality of *C. taeniops* is marked by blue pin. The circular mitochondrial genome of *C. taeniops* is represented and annotated using the MitoAnnotator online server. Different color arcs highlight the presence of PCGs, rRNAs, tRNAs, and CR. Species photograph was taken by Fantong Zealous Gietbong from the Ministry of Livestock, Fisheries and Animal Industries (MINEPIA), Yaounde, Cameroon. The linearized view of the complete mitochondrial genome organization reveals the duplication of *tRNA-Asp* (D) and CR in *C. argus* comparison with other *Cephalopholis* species.

In the current investigation, we elucidated the mitogenome of *C. taeniops*, revealing a length of 16,572 base pairs (bp) with GenBank accession no. OQ420715. Notably, the mitogenome of *C. taeniops* exhibited the shortest length among the *Cephalopholis* species, ranging from 16,585 bp (*Cephalopholis leopardus* and *Cephalopholis miniata*) to 16,771 bp (*Cephalopholis boenak*). Comprising 13 PCGs, 22 tRNAs, two rRNAs, and an AT-rich control region, the mitogenome of *C. taeniops* displayed a unique arrangement. The positive strand accommodated 12 PCGs, two rRNAs, and 14 tRNAs, while the negative strand housed *ND6* and 8 tRNAs (Table 1, Figure 1). The gene order in *C. taeniops*, along with other species (*C. boenak*, *C. leopardus*, *C. miniata*, *Cephalopholis sexmaculata*, *Cephalopholis sonnerati*, *Cephalopholis urodeta*, and *Cephalopholis spiloparaea*), mirrored that of the ancestral teleosts, with the exception of *Cephalopholis argus*, which exhibited content duplication of *trnD* and CR, as reported previously [41] (Figure 1).

The mitogenome of *C. taeniops* demonstrated an AT bias (54.99%), with nucleotide composition comprising 28.93% A, 26.06% T, 16.27% G, and 28.74% C. Similar AT biasness was observed in other *Cephalopholis* species, ranging from 54.99% (*C. taeniops*) to 56.94% (*C. boenak*). In the *C. taeniops* mitogenome, the AT skew and GC skew were calculated as 0.052 and −0.277, respectively. Comparative analysis with other *Cephalopholis* mitogenomes revealed an AT skew ranging from 0.026 (*C. argus*) to 0.061 (*C. sonnerati*) and a GC skew ranging from −0.282 (*C. leopardus* and *C. sonnerati*) to −0.247 (*C. argus*) (Table S2).

Our investigation further unveiled nine intergenic spacers totaling 64 bp and five overlapping regions spanning 23 bp in the *C. taeniops* mitogenome. The longest intergenic spacer (37 bp) was identified between *tRNA-Asn* (N) and *tRNA-Cys* (C), while the most extensive overlapping region (10 bp) occurred between ATP synthase 8 (*atp8*) and ATP synthase 6 (*atp6*) genes. A comparative analysis with other *Cephalopholis* mitogenomes disclosed intergenic spacer numbers ranging from 9 to 12, with the most extended spacer (37 bp to 40 bp) observed between *trnN* and *trnC* (Table S3). Moreover, six overlapping regions were consistently observed in most *Cephalopholis* mitogenomes, with a maximum length of 10 bp between *atp8* and *atp6*. Notably, an overlapping region (1 bp) between *atp6* and cytochrome c oxidase subunit III (*COIII*) was common in most *Cephalopholis* species but absent in *C. taeniops*. Additionally, an unconventional intergenic spacer (3 bp) was noted between *tRNA-Thr* (T) and *tRNA-Pro* (P) in *C. argus*, contrasting with the single base pair overlap present in the same gene boundary in other species (Table S3). The observed genetic variations within the mitogenomes of *Cephalopholis* species provide valuable insights into their evolutionary processes and energy metabolism, consistent with comparable findings in other fish species [58]. This investigation adds crucial information about the structural characteristics of *Cephalopholis* mitogenomes, thereby enhancing our comprehension of the functions encoded by these mitogenomes and their constituent genes.

### 2.2. Protein-Coding Genes

**Figure 2:**
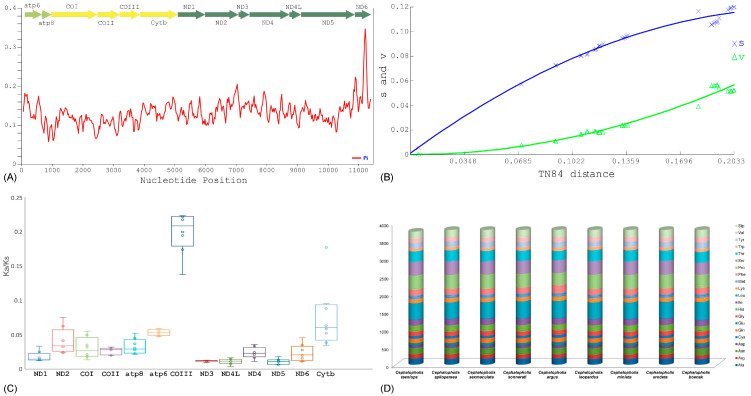
(**A**) Gene genetic diversity (Pi) of mitochondrial PCGs furnishing the genetic variations of *C. taeniops* and other *Cephalopholis* congeners. (**B**) Graph exhibits the relationship between transitions (s) and transversions (v) concerning genetic divergence of PCGs combining with all three codon positions, utilizing the Kimura 2-parameter (TN84) distance method with crosses indicating transition events and triangles representing transversion events. The curves delineate the non-saturated trends in the variance of transitions and transversions as genetic distance increases. (**C**) Box plot showing the pairwise divergence of Ka/Ks ratio for each one of the mitochondrial PCGs. (**D**) Abundance of codon usage of *Cephalopholis* species mitochondrial genomes including *C. taeniops*.

The mitogenome of *C. taeniops* comprises 13 PCGs, with the shortest length observed in *atp8* and the longest in *ND5*. The total length of *C. taeniops* PCGs is 11,301 base pairs (bp), constituting 68.19% of the complete mitogenome. In contrast to other *Cephalopholis* species, the total PCG length ranges from 11,301 bp (*C. taeniops*) to 11,430 bp (*C. argus*). Seven additional species (*C. boenak*, *C. leopardus*, *C. miniata*, *C. sexmaculata*, *C. sonnerati*, *C. urodeta*, and *C. spiloparaea*) maintain an equal PCG length of 11,429 bp in their mitogenomes (Table S2). The PCGs in *Cephalopholis* species exhibit an AT bias ranging from 54.57% (*C. taeniops*) to 56.7% (*C. argus*). AT skews and GC skews in *Cephalopholis* PCGs vary from −0.060 (*C. argus*) to −0.012 (*C. miniata* and *C. spiloparaea*) and −0.328 (*C. leopardus*) to −0.277 (*C. argus*). In the mitogenome of *C. taeniops*, most PCGs start with ATG, except for *COI* (GTG) and *atp6* (TTG). The *COI* gene initiates with GTG in all *Cephalopholis* species, while *atp6* starts with TTG in three species and CTG in six others. Notably, an exception is observed with *ND4* in *C. argus*, which starts with GTG instead of ATG. In *C. taeniops*, six PCGs terminate with TAA, *COIII* with GGC, and the remaining PCGs exhibit incomplete stop codons. An exception is *ND1* in *C. boenak*, *COI* in five species, *ND5* in *C. argus*, and *ND6* in two species, where TAG serves as the stop codon (Table S4). Nucleotide diversity analysis, using a sliding window approach on concatenated PCGs, yielded an average nucleotide diversity value (Pi) of 0.13191, with 3582 polymorphic sites across all *Cephalopholis* species (Figure 2A). A saturation analysis indicated non-saturation for both transitions and transversions with increasing Kimura 2-parameter (TN84) divergence values (Figure 2B).

### 2.3. Substitutions Pattern and Codon Usage

Darwinian selection stands as a pivotal hypothesis in elucidating the evolutionary dynamics of genes under positive selection, playing a crucial role in the divergence of species [59,60,61,62,63]. The examination of synonymous (Ks) and nonsynonymous (Ka) substitution rates within PCGs provides evidence for Darwinian selection and adaptive molecular evolution in vertebrates [64,65]. The Ka/Ks ratio serves as an established indicator of selective pressure and evolutionary relationships at the molecular level, applicable to both homogenous and heterogeneous species [66,67]. In this study, we investigated the evolutionary rates between homologous gene pairs by calculating Ka/Ks substitutions for *C. taeniops* and comparing them with *Cephalopholis* species. The Ka/Ks ratio ranged from 0.0109 ± 0.004 in *nad5* to 0.1986 ± 0.030 in *cox3* and the resulted following order: *nad5* < *nad4L* < *nad3* < *nad1* < *nad6* < *nad4* < *cox2* < *cox1* < *atp8* < *nad2* < *atp6* < *cytb* < *cox3* (Figure 2C). Most PCGs exhibited Ka/Ks values less than 1, indicating a strong negative selection among the studied *Cephalopholis* species, suggesting that the mutations were replaced by synonymous substitutions (Table S5). A comparative analysis of the Ka/Ks ratio among 13 PCGs of *Cephalopholis* species showed that all PCGs are evolving under negative selection (Table S6, Figure S1). This observation reflects the influence of natural selection in mitigating deleterious mutations with negative selective coefficients, aligning with general patterns observed in other vertebrates [65,66]. Thus, the comparative analysis of Ka/Ks within *Cephalopholis* species’ mitogenomes offers a platform for gaining insights into the nuances of natural selection shaping the evolutionary trajectory of species. This analysis aids in unraveling the intricate interplay between mutations and selective pressures, elucidating their collective role in steering the evolution of proteins. The codons corresponding to each amino acid were found to be conserved across all PCGs in the compared *Cephalopholis* species. An analysis of RSCU unveiled a maximal abundance of leucine, proline, serine, and threonine in the PCGs of *C. taeniops*, whereas aspartic acid, cysteine, glutamic acid, and tryptophan were less prevalent (Figure 2D). Similar amino acid abundance patterns were observed in other *Cephalopholis* species, mirroring those in *C. taeniops* (Table S7). Notably, the RSCU analysis demonstrated a significant decrease in the frequency of the ACG codon in threonine and the AGT codon in serine in *C. taeniops* (Figure S2). A comparative RSCU analysis further revealed a substantial reduction in the frequency of the GCG codon in alanine across most species (*C. argus*, *C. leopardus*, *C. miniata*, *C. sexmaculata*, *C. sonnerati*, *C. urodeta*, *C. spiloparaea*), except in *C. boenak* where ACT was observed in threonine (Table S7).

### 2.4. Ribosomal RNA and Transfer RNA Genes

The mitogenome of *C. taeniops* encompasses two ribosomal RNA molecules, namely, *12S rRNA* (953 bp) and *16S rRNA* (1712 bp), collectively contributing to 16.08% of the entire mitogenome. A comparative analysis with other *Cephalopholis* species revealed varying lengths of rRNAs, ranging from 2663 bp (*C. miniata* and *C. spiloparaea*) to 2679 bp (*C. argus*). The rRNA genes exhibit AT bias, ranging from 53.03% (*C. taeniops*) to 54.91% (*C. argus*) (Table S2). The AT skews and GC skews range from 0.184 (*C. argus*) to 0.263 (*C. boenak*) and −0.136 (*C. boenak*) to −0.084 (*C. argus*), respectively. In *C. taeniops*, the cumulative length of tRNA genes is 1565 bp, contributing 9.44% to the entire mitogenome. A comparative assessment with other *Cephalopholis* species shows the total tRNA length varying from 1562 bp (*C. sonnerati*) to 1638 bp (*C. argus*). The tRNA genes of *Cephalopholis* species display AT bias, ranging from 55.62% (*C. boenak*) to 57.63% (*C. argus*), with the AT skew ranging from 0.008 (*C. taeniops*) to 0.031 (*C. boenak*) (Table S2). Ribosomes, universally conserved ribonucleoproteins, facilitate the translation of genetic information from mRNAs to proteins. The structural arrangement of these rRNA genes, especially conserved loops, provides crucial insights into the catalytic processes underlying protein synthesis [68]. In *C. taeniops*, most tRNAs exhibit classic clover-leaf secondary structures, except for *trnS1* (GCT), which lacks the DHU stem (Figure S3). Wobble base pairings are observed in 18 tRNAs, with the highest number in *trnA* and *trnE* (Figure S3). These pairings occur in various tRNA regions, including the DHU stem, acceptor stem, TψC stem, and anticodon stem, indicating sites with G × U/T pairs for recognition by proteins and other RNAs. The anticodons of all 21 tRNAs exhibited uniformity across all *Cephalopholis* species, with the exception of *tRNA-Ser* (S1) (AGC), which was exclusively identified in three species, *C. taeniops*, *C. argus*, and *C. boenak*. Notably, a duplication event of *tRNA-Asp* (D) in *C. argus* resulted in both tRNAs featuring the GAC anticodon (Table S8). This allows wobble pairs to play essential roles in diverse biological processes [69,70]. tRNAs, acting as adaptor molecules, facilitate the translation of genetic information into protein sequences by delivering amino acids during translation. Additionally, gene rearrangements of tRNAs and high levels of length heteroplasmy in the WANCY region contribute to illuminating the evolution of mitochondrial genes [71,72].

### 2.5. Features of Control Region

**Figure 3:**
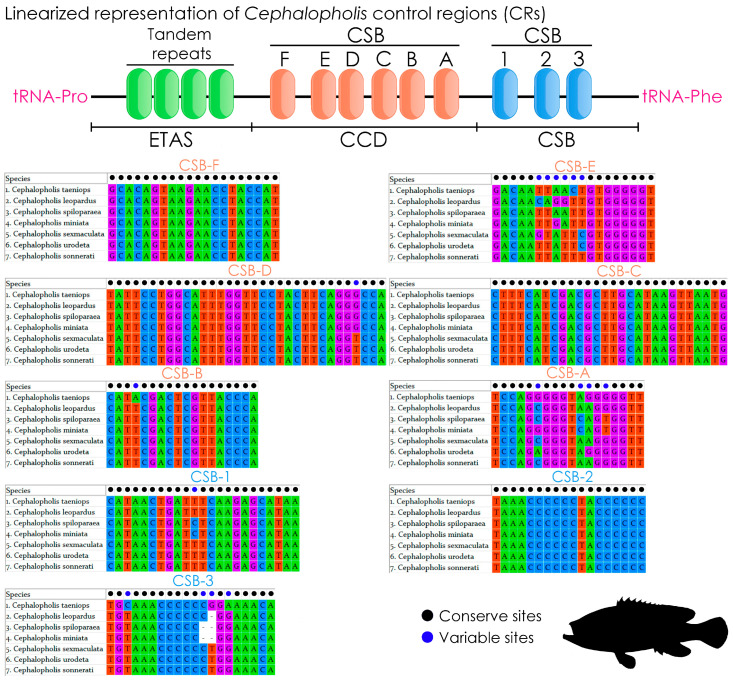
Schematic diagram shows the comparison of length and nucleotide composition of nine different conserved domains of *C. taeniops* and other six *Cephalopholis* congeners. The conserved and variable nucleotides are marked in black and blue circles. The gaps are denoted by dash symbol.

The CR of *C. taeniops* spans 873 base pairs (bp), representing 5.27% of the total mitogenome. A comparative analysis across various *Cephalopholis* species reveals a diverse range of CR lengths, ranging from 813 bp (*C. argus*) to 1064 bp (*C. boenak*). The CRs exhibit an AT bias, varying from 62.24% (*C. argus*) to 70.11% (*C. boenak*), with AT skews ranging from −0.035 (*C. boenak*) to 0.103 (*C. argus*) (Table S2). In scrutinizing the extant mitogenomes of six *Cephalopholis* species, a comprehensive examination of diverse domains was conducted, following the methodology outlined for *C. sonnerati* (KC593378) [41]. A comparative analysis reveals the presence of six central conserved domains (CCDs), CSB-A, CSB-B, CSB-C, CSB-D, CSB-E, and CSB-F, along with three conserved sequence blocks (CSBs), CSB-1, CSB-2, and CSB-3, within the CR of *C. taeniops* and the other six *Cephalopholis* species (*C. leopardus*, *C. miniata*, *C. sexmaculata*, *C. sonnerati*, *C. urodeta*, and *C. spiloparaea*). This observation aligns with patterns identified in other teleost mitogenomes [6,41]. Among the CCDs, CSB-D stands out as the longest at 33 base pairs, while CSB-A, CSB-B, CSB-C, CSB-E, and CSB-F exhibit lengths of 18 base pairs, 18 base pairs, 28 base pairs, 19 base pairs, and 20 base pairs, respectively (Figure 3). Comparative analyses unveil significant nucleotide variability within CSB-A and CSB-E (four and six base pairs, respectively), while other CCDs remain largely conserved across all *Cephalopholis* species. Within the CSBs, CSB-1 is the longest at 23 base pairs, with CSB-2 and CSB-3 exhibiting lengths of 18 base pairs and 20 base pairs, respectively (Figure 3). Comparative analyses of CSBs reveal notable nucleotide variability within CSB-3 (four base pairs), while CSB-1 and CSB-2 remain predominantly conserved. Due to unprecedented length variation and heteroplasmy, the CRs of *C. argus* (KC593377) and *C. boenak* (KC537759) were not investigated. The CR of *C. argus* lacks certain sequence elements found in other grouper species, shares no significant sequence similarity, and features an additional *tRNA-Asp* (D) insertion in the middle of the region. Except for *C. sexmaculata* (2.7 copy number of consensus 17 bp: CATATATGTATAGTAAC), no other *Cephalopholis* species’ CRs contain tandem repeats in the extended termination-associated sequences (ETAS) region. The repeat-rich extended termination-associated sequence (ETAS) region emerges as the most dynamically variable segment within the CR, characterized by specific motifs. This variability prompts the formation of stable hairpin loops, hypothesized to function as sequence-specific signals for the termination of mitochondrial DNA (mtDNA) replication [41]. Beyond the conserved attributes, the CRs of *Cephalopholis* species harbor highly polymorphic sequences, offering a robust tool for distinguishing between species and elucidating population structures in fish, a phenomenon well documented in other fish species, including groupers [73,74]. Furthermore, the intricate mechanisms governing the CR, such as genomic rearrangement through double replications, random loss, dimer-mitogenomes, and non-random loss, contribute significantly to comprehending the structural diversity of mitogenomes and the complexities inherent in the evolution of mitochondrial genomes.

### 2.6. Genetic Distances and Phylogenetic Relationship

**Figure 4:**
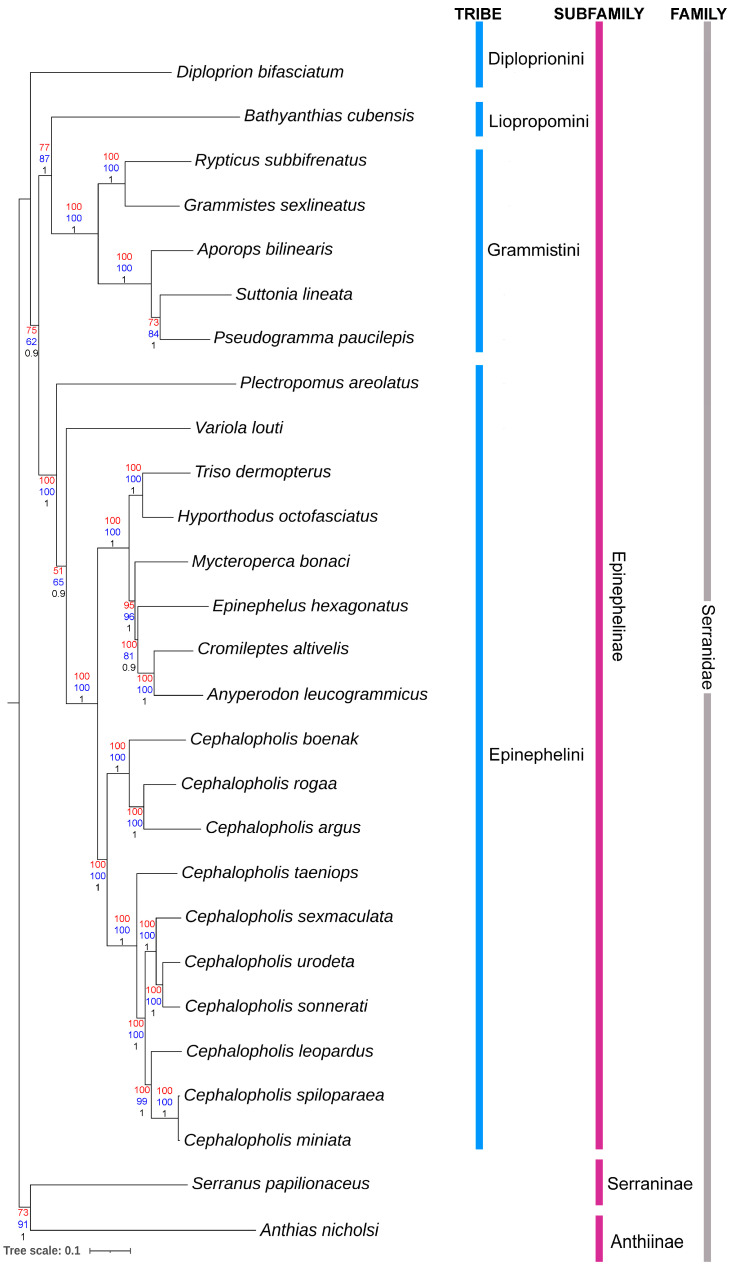
The maximum likelihood (ML) phylogeny constructed by 13 concatenated PCGs clearly discriminate *C. taeniops* from other *Cephalopholis* congeners. The cladistic pattern also elucidate insights into the evolutionary relationships of different taxonomic levels (tribe and subfamily) of fishes under the family Serranidae. ML bootstrap supports (red color), NJ bootstrap support (blue color), and Bayesian posterior probability values (black color) are indicated at each node, reflecting the statistical support for each branching point in the tree.

The serranids exhibit an overall mean genetic distance of 25.4% in the current mitogenomic dataset. Among the *Cephalopholis* species, inter-species genetic distances range from 0.06% (*C. miniata* and *C. spiloparaea*) to 20.8% (*C. argus* and *C. spiloparaea*). In the four tribes (Epinephelini, Diploprionini, Grammistini, and Liopropomini) within the subfamily Epinephelinae, genetic distances range from 28% to 30.9%. Furthermore, the subfamily Epinephelinae demonstrates genetic distances of 30.2% to 32.5% with other serranid subfamilies, Serraninae and Anthiinae, respectively. The maximum likelihood (ML) topology, constructed using concatenated protein-coding genes, distinctly separates all serranids, elucidating their phylogenetic relationships (Figure 4). Taxa from different taxonomic lineages, both at the tribe and subfamily levels, exhibit clear clustering patterns. *Cephalopholis* species display a cohesive and monophyletic clustering pattern in the cladistics analysis, aligning with previous evolutionary hypotheses on groupers [24,30]. Compelling evidence supports the consideration of *Cephalopholis* as a valid genus, as indicated by both the recent cladistic analysis based on mitogenomes and previous screenings using partial mitochondrial and nuclear genes [24,28,30]. Notably, the mitogenome of *Aethaloperca rogaa* clusters closely with *C. argus* and *C. boenak* in the ML phylogeny. The classification of the genus *Aethaloperca* is contentious, and *A. rogaa*, based on morphological features, was previously considered to belong to a monotypic genus [7]. However, recent studies propose synapomorphic characters that unite *Aethaloperca* with *Cephalopholis*, supporting its placement within the latter. The present mitogenome-based ML phylogeny corroborates this, designating *A. rogaa* nested under the *Cephalopholis* clade. Nevertheless, it is essential to subject this hypothesis to scrutiny through a comprehensive examination of additional morphological characteristics pertaining to these groupers and their related counterparts. Within *Cephalopholis* species, two subclusters emerge in the ML phylogeny. Three species (*C. argus*, *C. boenak*, and *C. rogaa*) cluster together, while the other seven species form a separate subclade. This subclustering does not seem to reflect any distributional distinction of these groupers in marine environments.

Additional studies on taxonomy and in-depth molecular data may provide further clarity on their evolutionary significance in marine ecosystems. Notably, the targeted species, *C. taeniops*, closely clusters with six other *Cephalopholis* species (*C. leopardus*, *C. miniata*, *C. sexmaculata*, *C. sonnerati*, *C. spiloparaea*, and *C. urodeta*) (Figure 4). Nevertheless, based on the cladding pattern, it can be asserted that *C. taeniops* serves as the ancestral species when compared to other closely related *Cephalopholis* species. In addition to the PCGs, the phylogenetic assessment of the studied serranids was reevaluated by incorporating both PCGs and rRNAs. rRNAs play a crucial role in protein synthesis, and their structures are highly conserved among different organisms [75]. Consequently, accounting for structural considerations becomes particularly important when aligning rRNA genes, especially for phylogenetic analyses, as such inferences necessitate comparisons of homologous characters across diverse sequences [76,77]. The NJ and BA topologies, based on 13 PCGs and 13 PCGs + two rRNAs, reveal a clustering pattern similar to that depicted by the concatenated PCGs alone (Figures S4–S6). Overall, this study underscores the effectiveness of mitochondrial genes in discriminating and elucidating the evolutionary relationships of serranids, as observed in other fish [78,79]. Large-scale genomic data offer valuable insights into time-calibrated phylogeny, adaptation to euryhalinity, and speciation [80]. Nevertheless, the adverse effects of global warming and extreme temperature events have impacted marine biodiversity, ecosystem functions, and services across all ocean basins over the past two decades, leading to significant losses in fisheries revenues and livelihoods in most maritime countries [81]. Recognizing the crucial role that genomic data play in conservation genetics and fish management [82], this study advocates for the generation of additional large-scale genomic data on groupers. This initiative aims to contribute to a more profound understanding of their evolution, diversification, and adaptation in marine environments.

## 3. Materials and Methods

### 3.1. Sampling and Species Identification

The *Cephalopholis* grouper specimen was obtained from the Atlantic Ocean, situated off the coast of Cameroon, Africa (Figure 1). Species identification was corroborated as *C. taeniops*, relying on morphological characteristics outlined in prior literature [7,11,12]. The body of the species is robust and slightly compressed, displaying a red-orange hue. It is adorned with numerous light blue spots, each surrounded by black margins, and the posterior part of all fins exhibits a slightly darker shade. The head and mouth are large, with a protruding lower jaw that features two robust canines at the anterior of each jaw. The upper arch boasts four knob-like undeveloped gill rakers and four fully developed ones, while the lower arch comprises three knob-like undeveloped gill rakers and 12 fully developed ones. The dorsal fin is continuous and equipped with nine spines, the anal fin features three spines, and the pelvic fin is supported by one spine. The posterior nostril is positioned close to the eye at the upper third level, while the anterior nostril is very close and slightly lower, furnished with a small flap. Muscle tissue from the ventral thoracic region was precisely excised and placed under sterile conditions within the Department of Marine Biology at Pukyong National University in Busan, Republic of Korea. Voucher specimens were accurately preserved in 10% formaldehyde at the Fisheries and Animal Industries (MINEPIA) facility in Yaoundé, Cameroon. Approval for the research protocol, granted by the Institutional Animal Care and Use Committee (IACUC) under the code PKNUIACUC-2022-72 on 15 December 2022, confirms that the use of biological material in the experiments adhered to ethical standards, ensuring that the targeted fish were not subjected to harm by the researchers. The range distribution of *C. taeniops* is mapped according to the IUCN data (.shp files) and the additional records published in the previous literature [7,10,11,12,13,14,15,16] (Figure 1).

### 3.2. DNA Extraction, Sequencing, and Assembly

The extraction of genomic DNA was conducted using the AccuPrep^®^ DNA extraction kit from Bioneer, situated in Daejeon, the Republic of Korea, following established standard protocols. The quality and quantity of the genomic DNA were thoroughly assessed using a NanoDrop spectrophotometer (Thermo Fisher Scientific D1000, Waltham, MA, USA). To obtain the complete mitogenome of *C. taeniops*, sequencing procedures were carried out on the NovaSeq platform at Macrogen (https://dna.macrogen.com/, accessed on 12 December 2023) in Daejeon, the Republic of Korea, facilitated by the Illumina platform. Sequencing libraries were prepared according to the manufacturer’s specifications for the TruSeq Nano DNA High-Throughput Library Prep Kit (Illumina, Inc., San Diego, CA, USA). In summary, 100 ng of genomic DNA underwent fragmentation utilizing adaptive focused acoustic technology (Covaris, Woburn, MA, USA), resulting in double-stranded DNA molecules with blunt ends and 5′-phosphorylation. Following the end-repair step, DNA fragments were size-selected using a bead-based method, modified with the addition of a single ‘A’ base, and ligated with TruSeq DNA UD Indexing adapters. The products were purified and enriched through PCR to generate the final DNA library. Library quantification was carried out using qPCR, following the qPCR Quantification Protocol Guide (KAPA Library Quantification Kits for Illumina Sequencing Platforms), and a quality assessment was performed using Agilent Technologies 4200 TapeStation D1000 screentape (Agilent Technologies, Santa Clara, CA, USA). Paired-end (2 × 150 bp) sequencing was conducted via Macrogen on the NovaSeq platform (Illumina, Inc., San Diego, CA, USA).

### 3.3. Mitogenome Assembly and Validation of Control Region

The processing of over 20 million raw reads was undertaken using the Cutadapt tool (http://code.google.com/p/cutadapt/, accessed on 12 December 2023) to trim adapters and remove low-quality bases with a Phred quality score (Q score) cutoff of 20. An assembly of the targeted genome from high-quality paired-end next-generation sequencing (NGS) reads was performed using Geneious Prime version 2023.0.1, employing reference mapping with the mitogenome of a closely related species as a reference and utilizing default mapping algorithms. To validate the mitogenome assembly, the alignment of overlapping regions was scrutinized using MEGA X [83]. The boundaries and orientations of individual genes were confirmed through the MITOS v806 (http://mitos.bioinf.uni-leipzig.de, accessed on 12 December 2023) and MitoAnnotator (http://mitofish.aori.u-tokyo.ac.jp/annotation/input/, accessed on 12 December 2023) web servers [84,85]. For the validation of protein-coding genes (PCGs), the translated putative amino acid sequences underwent analysis using the Open Reading Frame Finder web tool (https://www.ncbi.nlm.nih.gov/orffinder/, accessed on 12 December 2023), based on the vertebrate mitochondrial genetic code. Additionally, to confirm the full-length control region, a target-specific primer pair (5′-CGAGCACTAACCTTCCGACC-3′ and 5′-GGCTAAGCAAGGTGTCGTG-3′) was designed for further amplification. The PCR was conducted using the TaKaRa Verity Thermal Cycler with a 1X PCR buffer, 1 U Taq polymerase, 10 pmol primers, 2.5 mM dNTPs, and 1 µL template DNA. Purification of the PCR products was carried out using the AccuPrep^®^ PCR/Gel Purification Kit (Bioneer, Daejeon, Republic of Korea). Subsequently, the amplicons were subjected to amplification with the BigDye^®^ Terminator v3.1 Cycle Sequencing Kit (Applied Biosystems, Foster City, CA, USA) and sequenced in both directions utilizing the ABI PRISM 3730XL DNA analyzer available at Macrogen (https://dna.macrogen.com/, accessed on 12 December 2023), Daejeon, the Republic of Korea. The assembly of the control region with the complete mitogenome involved ensuring the alignment of overlapping regions through MEGA X, after eliminating any noisy segments via the SeqScanner version 1.0 (Applied Biosystems Inc., Foster City, CA, USA). The resulting *C. taeniops* mitogenome was appropriately submitted to the global GenBank database to acquire a unique accession number.

### 3.4. Characterization and Comparative Analyses

We utilized the MitoAnnotator (http://mitofish.aori.u-tokyo.ac.jp/annotation/input/, accessed on 12 December 2023) to construct a three-dimensional representation of the generated mitogenome. Our comprehensive comparative analysis aimed to evaluate the mitogenomic architecture and variations in our sequenced data in comparison to eight existing mitogenomes of *Cephalopholis* species [41,42,43,44,45,46] (Table S1). Due to taxonomic uncertainties found in Eschmeyer’s Catalog of Fishes, GenBank, and previous investigations [7,30], the mitochondrial genome of *Aethaloperca rogaa* (currently *Cephalopholis rogaa*) (KC593376) was excluded from the comparative analysis of structure and variation. Manually calculated values included the intergenic spacers between adjacent genes and overlapping regions. Nucleotide compositions within protein-coding genes (PCGs), rRNAs, tRNAs, and the control region (CR) were determined using MEGA X. A sliding window analysis of nucleotide diversity, with a window size of 200 bp and a step size of 25 bp, was conducted using DnaSP6.0 [86]. Base composition skews were computed using established formulas: AT-skew = [A − T]/[A + T] and GC-skew = [G − C]/[G + C] [87]. The saturation of the transition codon of the mitochondrial PCGs based on transition (s) and transversion (v), as well as AT and GC skews, was depicted using DAMBE6 [88]. The validation of the initiation and termination codons for each PCG, along with compliance with the vertebrate mitochondrial genetic code, was performed using MEGA X. The analysis involved the computation of relative synonymous codon usage (RSCU), the relative abundance of amino acids, and the distribution of codons using DnaSP6.0. Subsequently, pairwise tests for synonymous (Ks) and nonsynonymous (Ka) substitutions were conducted between *Cephalopholis taeniops* and other *Cephalopholis* species, employing DnaSP6.0. Additionally, the boundaries of rRNA and tRNA genes were confirmed through the utilization of the tRNAscan-SE Search Server 2.0 in conjunction with ARWEN 1.2 [89,90]. The identification of structural domains within the control region was achieved by conducting CLUSTAL X alignments, following previous research [41,91].

### 3.5. Genetic Distance and Phylogenetic Analyses

Pairwise genetic distances among various taxonomic levels within the Serranidae family were calculated using the Kimura 2-parameter (K2P) method within MEGA X. For the phylogenetic analysis, excluding *Cephalopholis* species, 16 mitogenomes from representative species across four tribes of the subfamily Epinephelinae (Epinephelini, Diploprionini, Grammistini, and Liopropomini) were retrieved from GenBank [6,41,46,92,93,94] (Table S1). *Anthias nicholsi* (OP056908) from the subfamily Anthiinae and *Serranus papilionaceus* (OK054500) from the subfamily Serraninae were designated as outgroup taxa [94,95]. Two concatenated datasets (13 PCGs and 13 PCGs + two rRNAs) were assembled using the iTaxoTools 0.1 tool to elucidate the primary evolutionary relationships among serranids, with a particular focus on *Cephalopholis* species within the subfamily Epinephelinae and tribe Epinephelini [96]. The neighbor-joining (NJ) phylogeny was constructed using the PCGs dataset and the K2P model through MEGA X. Model selection analysis identified the ‘GTR + G + I’ model as the most suitable, serving as the optimal model for all PCGs and yielding the lowest Bayesian information criterion (BIC) scores. This model selection process was conducted through PartitionFinder 2 on the CIPRES Science Gateway v3.3 and JModelTest v2 [97,98,99]. The maximum likelihood (ML) phylogeny was constructed using the IQ-Tree web server with 1000 bootstrap samples and PhyML 3.0, following the standard protocol [100,101]. A Bayesian (BA) tree was constructed using Mr. Bayes 3.1.2, with nst = 6, involving one cold and three hot metropolis-coupled Markov chain Monte Carlo (MCMC) chains. The analysis ran for 10,000,000 generations, with tree sampling at every 100th generation, and 25% of the samples were discarded as burn-in [102]. The resulting BA tree was visualized using the iTOL v4 web server (https://itol.embl.de/login.cgi, accessed on 12 December 2023) [103].

## 4. Conclusions

The escalating impacts of global warming and extreme temperature events pose a threat to marine biodiversity, resulting in considerable losses in fisheries worldwide. Groupers (Perciformes: Serranidae) are economically and recreationally valuable reef-associated fish. Beside species invention, the understanding of evolutionary patterns in groupers based on mitogenomes is currently limited on a global scale. This research extensively delves into the structure of and variations in Epinephelinae mitogenomes, emphasizing the complete mitogenome of *Cephalopholis taeniops* from the Eastern Atlantic Ocean. The study reveals substantial genetic divergence between *C. taeniops* and its congeners, providing crucial insights into the evolutionary dynamics of groupers. These findings offer valuable resources for further investigations into grouper species identification, conservation genetics, speciation, and other evolutionary biology studies. The acquired genetic knowledge is pivotal for formulating effective conservation strategies in MPAs, safeguarding species diversity, and ensuring the sustainability of marine life, especially within the Serranidae family.

## Figures and Tables

**Table 1:** List of annotated mitochondrial genes, boundaries, size, and intergenic nucleotides of *Cephalopholis taeniops*.

Genes	Start	Stop	Strand	Size (bp)	Intergenic Nucleotide	Anticodon	Start Codon	Stop Codon
*tRNA-Phe* (F)	1	70	H	70	0	TTC	.	.
*12S rRNA*	71	1023	H	953	0	.	.	.
*tRNA-Val* (V)	1024	1095	H	72	0	GTA	.	.
*16S rRNA*	1096	2807	H	1712	0	.	.	.
*tRNA-Leu* (L2)	2808	2882	H	75	0	TTA	.	.
*ND1*	2883	3857	H	975	5	.	ATG	TAA
*tRNA-Ile* (I)	3863	3932	H	70	−1	ATC	.	.
*tRNA-Gln* (Q)	3932	4002	L	71	0	CAA	.	.
*tRNA-Met* (M)	4003	4072	H	70	0	ATG	.	.
*ND2*	4073	5118	H	1046	0	.	ATG	TA-
*tRNA-Trp* (W)	5119	5189	H	71	1	TGA	.	.
*tRNA-Ala* (A)	5191	5259	L	69	0	GCA	.	.
*tRNA-Asn* (N)	5260	5332	L	73	37	AAC	.	.
*tRNA-Cys* (C)	5370	5437	L	68	0	TGC	.	.
*tRNA-Tyr* (Y)	5438	5508	L	71	1	TAC	.	.
*COI*	5510	7060	H	1551	0	.	GTG	TAA
*tRNA-Ser* (S2)	7061	7131	L	71	1	TCA	.	.
*tRNA-Asp* (D)	7133	7205	H	73	8	GAC	.	.
*COII*	7214	7904	H	691	0	.	ATG	T--
*tRNA-Lys* (K)	7905	7977	H	73	1	AAA	.	.
*ATP8*	7979	8146	H	168	−10	.	ATG	TAA
*ATP6*	8137	8819	H	683	0	.	TTG	TA-
*COIII*	8820	9604	H	785	0	.	ATG	TA-
*tRNA-Gly* (G)	9605	9676	H	72	0	GGA	.	.
*ND3*	9677	10,025	H	349	0	.	ATG	T--
*tRNA-Arg* (R)	10,026	10,094	H	69	0	CGA	.	.
*ND4L*	10,095	10,391	H	297	−7	.	ATG	TAA
*ND4*	10,385	11,765	H	1381	0	.	ATG	T--
*tRNA-His* (H)	11,766	11,835	H	70	0	CAC	.	.
*tRNA-Ser* (S1)	11,836	11,907	H	72	6	AGC	.	.
*tRNA-Leu* (L1)	11,914	11,986	H	73	0	CTA	.	.
*ND5*	11,987	13,825	H	1839	−4	.	ATG	TAA
*ND6*	13,822	14,343	L	522	0	.	ATG	TAA
*tRNA-Glu* (E)	14,344	14,412	L	69	4	GAA	.	.
*Cyt b*	14,417	15,557	H	1141	0	.	ATG	T--
*tRNA-Thr* (T)	15,558	15,630	H	73	−1	ACA	.	.
*tRNA-Pro* (P)	15,630	15,699	L	70	0	CCA	.	.
*Control region* (CR)	15,700	16,572	H	873	.	.	.	.

## Data Availability

The genome sequence data that support the findings of this study are openly available in GenBank of NCBI at https://www.ncbi.nlm.nih.gov, under the accession no. OQ420715.

## Supplementary Materials

The following supporting information can be downloaded at: https://www.mdpi.com/article/10.3390/ijms25031822/s1.
