# Supplementary material for: Mitogenomic Characterization and Phylogenetic Placement of African Hind, Cephalopholis taeniops: Shedding Light on the Evolution of Groupers (Serranidae: Epinephelinae)
Source: Int J Mol Sci. 2024 Feb 2;25(3):1822. doi: 10.3390/ijms25031822 (PMC10855530; doi:10.3390/ijms25031822)
Supplement: Supplementary file 1 [file ijms-25-01822-s001.zip › ijms-2809025-supplementary.pdf]

Supporting information

Table S1. Details on the mitogenomes generated during this study and those available in GenBank for *Cephalopholis* and other Serranid species.

Sl. No.	Subfamily	Tribe	Species	Accession No.	References
1	Epinephelinae	Epinephelini	*Cephalopholis taeniops*	OQ420715	This study
2			*Cephalopholis argus*	KC593377	Zhuang et al. 2013 [41]
3			*Cephalopholis boenak*	KC537759	Li et al. 2014 [42]
4			*Cephalopholis leopardus*	MW560467	Wang et al. 2022 [46]
5			*Cephalopholis miniata*	MW423580	Meng et al. 2021 [45]
6			*Cephalopholis sexmaculata*	KJ469385	Hsiao et al. 2016 [43]
7			*Cephalopholis sonnerati*	KC593378	Zhuang et al. 2013 [41]
8			*Cephalopholis urodeta*	KU891818	Guo et al. 2016 [44]
9			*Cephalopholis spiloparaea*	MW560468	Wang et al. 2022 [46]
10			*Aethaloperca/Cephalopholis rogaa*	KC593376	Zhuang et al. 2013 [41]
11			*Epinephelus hexagonatus*	MW560469	Wang et al. 2022 [46]
12			*Anyperodon leucogrammicus*	GQ131336	GenBank
13			*Cromileptes altivelis*	KC593375	Zhuang et al. 2013 [41]
14			*Hyporthodus octofasciatus*	JX135579	Zhuang et al. 2013 [41]
15			*Mycteroperca bonaci*	OP035077	Hoban et al. 2022 [64]
16			*Plectropomus areolatus*	KC262636	Shen et al. 2013 [62]
17			*Triso dermopterus*	KC593371	Zhuang et al. 2013 [41]
18			*Variola louti*	KC593369	Zhuang et al. 2013 [41]
19		Diploprionini	*Diploprion bifasciatum*	KP256530	Wang et al. 2016 [63]
20		Grammistini	*Aporops bilinearis*	OP035136	Hoban et al. 2022 [64]
21			*Grammistes sexlineatus*	AP017437	Satoh et al. 2016 [6]
22			*Pseudogramma paucilepis*	OR546228	Hoban et al. 2022 [64]
23			*Rypticus subbifrenatus*	OP056927	Hoban et al. 2022 [64]
24			*Suttonia lineata*	OP035248	Hoban et al. 2022 [64]
25		Liopropomini	*Bathyanthias cubensis*	OR546196	Hoban et al. 2022 [64]
26	Anthiinae		*Anthias nicholsi*	OP056908	Hoban et al. 2022 [64]
27	Serraninae		*Serranus papilionaceus*	OK054500	Vella et al. 2022 [65]

Table S2. Nucleotide composition of the mitochondrial genome in different *Cephalopholis* species.

Species Name	Size (bp)	A%	T%	G%	C%	A+T%	AT-Skew	GC-Skew
**Complete mitogenome**								
C. taeniops	16572	28.93	26.06	16.27	28.74	54.99	0.0523	-0.2768
C. argus	16767	29.16	27.69	16.24	26.91	56.85	0.0258	-0.2473
C. boenak	16771	29.58	27.36	15.84	27.22	56.94	0.0389	-0.2644
C. leopardus	16585	29.16	25.97	16.10	28.76	55.13	0.0579	-0.2821
C. miniata	16585	29.21	26.07	16.18	28.54	55.28	0.0568	-0.2764
C. sexmaculata	16589	29.35	26.01	16.08	28.56	55.36	0.0604	-0.2795
C. sonnerati	16587	29.62	26.21	15.85	28.32	55.83	0.0610	-0.2823
C. urodeta	16592	29.46	26.18	16.00	28.36	55.64	0.0589	-0.2788
C. spiloparaea	16587	29.23	26.06	16.15	28.56	55.29	0.0572	-0.2778
**PCGs**								
C. taeniops	11301	26.69	27.88	15.37	30.06	54.57	-0.0219	-0.3233
C. argus	11430	26.65	30.05	15.66	27.64	56.70	-0.0600	-0.2766
C. boenak	11429	27.39	29.19	15.14	28.29	56.58	-0.0319	-0.3028
C. leopardus	11429	27.02	27.74	15.21	30.04	54.76	-0.0131	-0.3278
C. miniata	11429	27.12	27.80	15.41	29.68	54.92	-0.0124	-0.3165
C. sexmaculata	11429	27.01	27.82	15.37	29.79	54.83	-0.0148	-0.3193
C. sonnerati	11429	27.36	28.10	15.05	29.49	55.46	-0.0134	-0.3242
C. urodeta	11429	27.20	27.99	15.15	29.65	55.19	-0.0143	-0.3236
C. spiloparaea	11429	27.11	27.77	15.39	29.73	54.88	-0.0121	-0.3178
**rRNAs**								
C. taeniops	2665	32.35	20.68	21.01	25.97	53.03	0.2201	-0.1054
C. argus	2679	32.51	22.40	20.64	24.45	54.91	0.1842	-0.0844
C. boenak	2666	33.12	21.12	20.48	25.28	54.24	0.2626	-0.1361

C. leopardus	2666	32.48	20.48	21.08	25.96	52.96	0.2266	-0.1037
C. miniata	2663	32.48	20.84	21.18	25.50	53.32	0.2183	-0.0925
C. sexmaculata	2668	32.68	20.84	20.95	25.52	53.52	0.2212	-0.0984
C. sonnerati	2671	32.65	21.15	20.97	25.23	53.80	0.2136	-0.0924
C. urodeta	2673	32.70	21.25	20.95	25.10	53.95	0.2122	-0.0902
C. spiloparaea	2663	32.56	20.84	21.14	25.46	53.40	0.2194	-0.0927
**tRNAs**								
C. taeniops	1565	28.43	27.99	23.26	20.32	56.42	0.0079	0.0674
C. argus	1638	29.24	28.39	22.59	19.78	57.63	0.0148	0.0662
C. boenak	1566	28.67	26.95	22.99	21.39	55.62	0.0310	0.0359
C. leopardus	1563	28.98	27.51	23.16	20.35	56.49	0.0260	0.0647
C. miniata	1565	29.07	27.80	23.07	20.06	56.87	0.0224	0.0696
C. sexmaculata	1565	28.88	27.67	23.26	20.19	56.55	0.0214	0.0705
C. sonnerati	1562	29.00	27.78	23.18	20.04	56.78	0.0214	0.0725
C. urodeta	1566	28.80	27.91	23.31	19.99	56.71	0.0157	0.0767
C. spiloparaea	1565	29.07	27.80	23.07	20.06	56.87	0.0224	0.0696
**CRs**								
C. taeniops	873	32.99	30.70	15.46	20.85	63.69	0.0359	-0.1483
C. argus	813	34.32	27.92	13.28	24.48	62.24	0.1027	-0.2964
C. boenak	1064	33.83	36.28	11.84	18.05	70.11	-0.0349	-0.2075
C. leopardus	880	33.75	30.45	14.43	21.36	64.20	0.0513	-0.1937
C. miniata	878	32.57	31.21	14.81	21.41	63.78	0.0214	-0.1824
C. sexmaculata	877	33.98	31.58	13.80	20.64	65.56	0.0365	-0.1987
C. sonnerati	878	34.51	30.98	13.90	20.62	65.49	0.0539	-0.1947
C. urodeta	877	34.78	30.22	14.82	20.18	65.00	0.0701	-0.1531
C. spiloparaea	880	32.61	31.48	14.66	21.25	64.09	0.0177	-0.1835

Table S3. Intergenic nucleotides of different *Cephalopholis* species. The duplication of tRNA and CR of *C. argus* are highlighted in gray colour.

**Genes**	***C. taeniops***	***C. argus***	***C. boenak***	***C. leopardus***	***C. miniata***	***C. sexmaculata***	***C. sonnerati***	***C. urodeta***	***C. spiloparaea***
tRNA-Phe (F)	0	0	0	0	0	0	0	0	0
12S rRNA	0	0	0	0	0	0	0	0	0
tRNA-Val (V)	0	1	0	0	0	1	1	1	0
16S rRNA	0	0	0	0	0	0	0	0	0
tRNA-Leu (L2)	0	0	0	0	0	0	0	0	0
ND1	5	4	4	5	5	7	6	6	5
tRNA-Ile (I)	-1	-1	-1	-1	-1	-2	-2	-2	-1
tRNA-Gln (Q)	0	0	0	0	0	0	0	0	0
tRNA-Met (M)	0	0	0	0	0	0	0	0	0
ND2	0	0	0	0	0	0	0	0	0
tRNA-Trp (W)	1	1	1	1	1	1	1	1	1
tRNA-Ala (A)	0	0	0	0	1	0	0	0	1
tRNA-Asn (N)	37	39	40	39	38	39	39	39	38
tRNA-Cys (C)	0	0	0	0	0	0	0	0	0
tRNA-Tyr (Y)	1	1	1	1	1	1	1	1	1
COI	0	0	0	0	1	0	0	0	1
tRNA-Ser (S2)	1	4	3	3	3	3	3	3	3
tRNA-Asp (D)	8	8	8	8	9	8	8	8	9
COII	0	0	0	0	0	0	0	0	5
tRNA-Lys (K)	1	1	1	1	1	1	1	1	1
ATP8	-10	-10	-10	-10	-10	-10	-10	-10	-10
ATP6	0	-1	-1	-1	-1	-1	-1	-1	-1
COIII	0	0	0	0	0	0	0	0	0
tRNA-Gly (G)	0	0	0	0	0	0	0	0	0
ND3	0	0	0	0	0	0	0	0	0
tRNA-Arg (R)	0	0	0	0	0	0	0	0	0
ND4L	-7	-7	-7	-7	-7	-7	-7	-7	-7

ND4	0	0	0	0	0	0	0	0	0
tRNA-His (H)	0	0	1	0	0	0	0	0	0
tRNA-Ser (S1)	6	6	7	9	20	9	8	9	10
tRNA-Leu (L1)	0	0	0	0	0	0	0	0	0
ND5	-4	-4	-4	-4	-4	-4	-4	-4	-4
ND6	0	0	0	0	0	0	0	0	0
tRNA-Glu (E)	4	6	6	4	4	4	4	4	4
Cyt b	0	0	0	0	0	10	0	0	0
tRNA-Thr (T)	-1	3	-1	-1	-1	-1	-1	-1	-1
tRNA-Pro (P)	0	0	0	0	0	0	0	0	0
Control region	-	0	-	-	-	-	-	-	-
tRNA-Asp (D)	-	0	-	-	-	-	-	-	-
Control region	-	-	-	-	-	-	-	-	-

Table S4. Comprehensive comparison of the start and stop codons of the PCGs across nine *Cephalopholis* mitogenomes.

	C. taeniops		***C. argus***		***C. boenak***		***C. leopardus***		***C. miniata***		***C. sexmaculata***		***C. sonnerati***		***C. urodeta***		***C. spiloparaea***	
**Genes**	Start	Stop	Start	Stop	Start	Stop	Start	Stop	Start	Stop	Start	Stop	Start	Stop	Start	Stop	Start	Stop
ND1	ATG	TAA	ATG	TAA	ATG	TAG	ATG	TAA	ATG	TAA	ATG	TAA	ATG	TAA	ATG	TAA	ATG	TAA
ND2	ATG	TA-	ATG	TA-	ATG	TA-	ATG	TA-	ATG	TA-	ATG	TA-	ATG	TA-	ATG	TA-	ATG	TA-
COI	GTG	TAA	GTG	TAA	GTG	TAA	GTG	TAG	GTG	TAG	GTG	TAA	GTG	TAG	GTG	TAG	GTG	TAG
COII	ATG	T--	ATG	T--	ATG	T--	ATG	T--	ATG	T--	ATG	T--	ATG	T--	ATG	T--	ATG	T--
ATP8	ATG	TAA	ATG	TAA	ATG	TAA	ATG	TAA	ATG	TAA	ATG	TAA	ATG	TAA	ATG	TAA	ATG	TAA
ATP6	TTG	TA-	TTG	TAA	CTG	TA-	CTG	TA-	GTA	TA-	CTG	TA-	CTG	TA-	CTG	TA-	GTA	TA-
COIII	ATG	TA-	ATG	TA-	ATG	TA-	ATG	TA-	ATG	TA-	ATG	TA-	ATG	TA-	ATG	TA-	ATG	TA-
ND3	ATG	T--	ATG	T--	ATG	T--	ATG	T--	ATG	T--	ATG	T--	ATG	T--	ATG	T--	ATG	T--
ND4L	ATG	TAA	ATG	TAA	ATG	TAA	ATG	TAA	ATG	TAA	ATG	TAA	ATG	TAA	ATG	TAA	ATG	TAA
ND4	ATG	T--	GTG	T--	ATG	T--	ATG	T--	ATG	T--	ATG	T--	ATG	T--	ATG	T--	ATG	T--
ND5	ATG	TAA	ATG	TAG	ATG	TAA	ATG	TAA	ATG	TAA	ATG	TAA	ATG	TAA	ATG	TAA	ATG	TAA
ND6	ATG	TAA	ATG	TAA	ATG	TAG	ATG	TAG	ATG	TAA	ATG	TAA	ATG	TAA	ATG	TAA	ATG	TAA
Cyt b	ATG	T--	ATG	TA-	ATG	T--	ATG	T--	ATG	T--	ATG	T--	ATG	T--	ATG	T--	ATG	T--

Table S5. Ka/Ks ratios of 13 PCGs in *Cephalopholis* mitogenomes.

Genes	*nad1*	*nad2*	*cox1*	*cox2*	*atp8*	*atp6*	*cox3*	*nad3*	*nad4l*	*nad4*	*nad5*	*nad6*	*cytb*
	0.0250	0.0622	0.0497	0.0288	0.0519	0.0590	0.2231	0.0126	0.0127	0.0167	0.0180	0.0455	0.0878
	0.0332	0.0747	0.0352	0.0197	0.0368	0.0470	0.1373	0.0112	0.0035	0.0110	0.0131	0.0349	0.0631
	0.0124	0.0323	0.0181	0.0288	0.0248	0.0578	0.2173	0.0106	0.0075	0.0242	0.0072	0.0200	0.0580
	0.0133	0.0238	0.0190	0.0224	0.0217	0.0540	0.1994	0.0127	0.0112	0.0357	0.0066	0.0113	0.0385
	0.0163	0.0411	0.0550	0.0319	0.0448	0.0531	0.2214	0.0099	0.0165	0.0252	0.0135	0.0214	0.1768
	0.0131	0.0326	0.0317	0.0278	0.0304	0.0518	0.2224	0.0121	0.0118	0.0212	0.0115	0.0276	0.0951
	0.0155	0.0349	0.0135	0.0306	0.0277	0.0478	0.1736	0.0130	0.0147	0.0215	0.0107	0.0157	0.0520
	0.0133	0.0238	0.0215	0.0200	0.0227	0.0562	0.1943	0.0125	0.0112	0.0333	0.0066	0.0108	0.0346
Average	0.0178	0.0407	0.0305	0.0262	0.0326	0.0533	0.1986	0.0118	0.0112	0.0236	0.0109	0.0234	0.0757
STDEV	0.0074	0.0183	0.0154	0.0048	0.0110	0.0044	0.0303	0.0011	0.0041	0.0081	0.0040	0.0121	0.0461

Table S6. Comparative pairwise Ka/Ks values of 13 PCGs in *Cephalopholis* species.

**Genes**	*C. taeniops/ C. argus*	*C. taeniops/ C. boenak*	*C. taeniops/ C. leopardus*	*C. taeniops/ C. miniata*	*C. taeniops/ C. sexmaculata*	*C. taeniops/ C. sonnerati*	*C. taeniops/ C. urodeta*	*C. taeniops/ C. spiloparaea*
nad1	0.025029104	0.033194589	0.012434062	0.013322884	0.016261498	0.01308699	0.015485687	0.013322884
nad2	0.062201908	0.074658754	0.032314241	0.023833844	0.041117389	0.0326344	0.034874546	0.023752969
cox1	0.01258958	0.011191443	0.010632642	0.01266055	0.00986701	0.012076173	0.013030404	0.012522686
cox2	0.012707444	0.003498757	0.007529225	0.01119403	0.016514708	0.011847849	0.01474869	0.01119403
atp8	0.087820622	0.063063063	0.057996051	0.038499506	0.176794061	0.095146493	0.051997359	0.034566807
atp6	0.045542448	0.034940835	0.019980162	0.01128509	0.021444847	0.027570887	0.015741834	0.01080789
cox3	0.016678433	0.010965613	0.024247788	0.035714286	0.025190964	0.021210714	0.021489672	0.033311702
nad3	0.049661926	0.035195848	0.01809816	0.018985821	0.055023923	0.031685166	0.013462202	0.021517091
nad4l	0.051907442	0.03681592	0.024815206	0.021702324	0.044790193	0.030445969	0.027712724	0.022687047
nad4	0.028816249	0.019676885	0.028760331	0.022401991	0.031939414	0.027777778	0.030569862	0.019997015
nad5	0.058957858	0.047048594	0.057809507	0.053991031	0.053102509	0.051792081	0.047751765	0.056217755
nad6	0.223081571	0.13734983	0.217318008	0.199376947	0.221386647	0.22238806	0.17361717	0.194297782
cytb	0.01803141	0.013130471	0.007202216	0.00655616	0.013473396	0.011466575	0.010734177	0.006646601

Table S7. The abundance of amino acids and RSCU value of the complete PCGs of *Cephalopholis* species.

Cephalopholis argus											
Codon	Count	RSCU	Codon	Count	RSCU	Codon	Count	RSCU	Codon	Count	RSCU
UUU-F	125	1.14	UCU-S	99	1.65	UAU-Y	86	1.08	UGU-C	22	0.54
UUC-F	94	0.86	UCC-S	65	1.08	UAC-Y	73	0.92	UGC-C	59	1.46
UUA-L	85	1.06	UCA-S	64	1.07	UAA-*	74	1.4	UGA-W	58	1.3
UUG-L	63	0.78	UCG-S	27	0.45	UAG-*	57	1.08	UGG-W	31	0.7
CUU-L	114	1.42	CCU-P	152	1.76	CAU-H	91	1.13	CGU-R	25	0.91
CUC-L	67	0.83	CCC-P	87	1.01	CAC-H	70	0.87	CGC-R	36	1.31
CUA-L	90	1.12	CCA-P	79	0.91	CAA-Q	84	1.33	CGA-R	27	0.98
CUG-L	64	0.8	CCG-P	28	0.32	CAG-Q	42	0.67	CGG-R	22	0.8
AUU-I	113	1.22	ACU-T	86	1.28	AAU-N	87	0.98	AGU-S	32	0.53
AUC-I	73	0.78	ACC-T	70	1.04	AAC-N	90	1.02	AGC-S	73	1.22
AUA-M	63	1.19	ACA-T	84	1.25	AAA-K	73	1.28	AGA-*	31	0.59
AUG-M	43	0.81	ACG-T	28	0.42	AAG-K	41	0.72	AGG-*	49	0.93
GUU-V	45	1.33	GCU-A	71	1.54	GAU-D	40	0.99	GGU-G	34	1.07
GUC-V	27	0.8	GCC-A	62	1.35	GAC-D	41	1.01	GGC-G	37	1.17
GUA-V	38	1.13	GCA-A	41	0.89	GAA-E	53	1.28	GGA-G	28	0.88
GUG-V	25	0.74	GCG-A	10	0.22	GAG-E	30	0.72	GGG-G	28	0.88
Cephalopholis boenak											
UUU-F	91	1.04	UCU-S	83	1.49	UAU-Y	96	1.06	UGU-C	27	0.72
UUC-F	84	0.96	UCC-S	74	1.33	UAC-Y	85	0.94	UGC-C	48	1.28
UUA-L	91	1.06	UCA-S	57	1.02	UAA-*	73	1.36	UGA-W	52	1.22
UUG-L	64	0.75	UCG-S	23	0.41	UAG-*	56	1.05	UGG-W	33	0.78
CUU-L	119	1.39	CCU-P	167	1.77	CAU-H	75	1.02	CGU-R	24	0.97
CUC-L	81	0.94	CCC-P	90	0.95	CAC-H	72	0.98	CGC-R	31	1.25
CUA-L	103	1.2	CCA-P	85	0.9	CAA-Q	74	1.21	CGA-R	25	1.01
CUG-L	57	0.66	CCG-P	35	0.37	CAG-Q	48	0.79	CGG-R	19	0.77

AUU-I	108	1.26	ACU-T	110	1.41	AAU-N	93	0.97	AGU-S	26	0.47
AUC-I	64	0.74	ACC-T	78	1	AAC-N	98	1.03	AGC-S	72	1.29
AUA-M	63	1.19	ACA-T	102	1.31	AAA-K	77	1.39	AGA-*	35	0.65
AUG-M	43	0.81	ACG-T	21	0.27	AAG-K	34	0.61	AGG-*	50	0.93
GUU-V	46	1.32	GCU-A	61	1.48	GAU-D	35	0.91	GGU-G	28	0.86
GUC-V	29	0.83	GCC-A	52	1.26	GAC-D	42	1.09	GGC-G	33	1.02
GUA-V	54	1.55	GCA-A	40	0.97	GAA-E	52	1.33	GGA-G	32	0.98
GUG-V	10	0.29	GCG-A	12	0.29	GAG-E	26	0.67	GGG-G	37	1.14
Cephalopholis taeniops											
UUU-F	94	1.14	UCU-S	96	1.51	UAU-Y	71	0.99	UGU-C	25	0.79
UUC-F	71	0.86	UCC-S	67	1.05	UAC-Y	73	1.01	UGC-C	38	1.21
UUA-L	77	1	UCA-S	71	1.12	UAA-*	66	1.31	UGA-W	47	1.11
UUG-L	42	0.55	UCG-S	38	0.6	UAG-*	50	1	UGG-W	38	0.89
CUU-L	108	1.4	CCU-P	175	1.76	CAU-H	88	1.02	CGU-R	27	0.91
CUC-L	94	1.22	CCC-P	110	1.11	CAC-H	84	0.98	CGC-R	43	1.45
CUA-L	86	1.12	CCA-P	80	0.8	CAA-Q	85	1.35	CGA-R	25	0.84
CUG-L	55	0.71	CCG-P	33	0.33	CAG-Q	41	0.65	CGG-R	24	0.81
AUU-I	103	1.23	ACU-T	109	1.42	AAU-N	87	0.98	AGU-S	28	0.44
AUC-I	65	0.77	ACC-T	78	1.02	AAC-N	90	1.02	AGC-S	82	1.29
AUA-M	52	0.95	ACA-T	87	1.13	AAA-K	75	1.39	AGA-*	29	0.58
AUG-M	57	1.05	ACG-T	33	0.43	AAG-K	33	0.61	AGG-*	56	1.11
GUU-V	28	0.91	GCU-A	53	1.23	GAU-D	23	0.62	GGU-G	37	1.21
GUC-V	33	1.07	GCC-A	74	1.71	GAC-D	51	1.38	GGC-G	28	0.92
GUA-V	39	1.27	GCA-A	37	0.86	GAA-E	55	1.41	GGA-G	32	1.05
GUG-V	23	0.75	GCG-A	9	0.21	GAG-E	23	0.59	GGG-G	25	0.82
Cephalopholis leopardus											
UUU-F	73	0.9	UCU-S	97	1.55	UAU-Y	76	1.03	UGU-C	24	0.64
UUC-F	90	1.1	UCC-S	66	1.05	UAC-Y	72	0.97	UGC-C	51	1.36
UUA-L	72	0.91	UCA-S	62	0.99	UAA-*	72	1.37	UGA-W	53	1.25
UUG-L	40	0.51	UCG-S	37	0.59	UAG-*	54	1.03	UGG-W	32	0.75

CUU-L	109	1.38	CCU-P	169	1.67	CAU-H	85	0.99	CGU-R	30	1.05
CUC-L	83	1.05	CCC-P	117	1.15	CAC-H	86	1.01	CGC-R	34	1.19
CUA-L	113	1.43	CCA-P	87	0.86	CAA-Q	77	1.25	CGA-R	23	0.81
CUG-L	57	0.72	CCG-P	33	0.33	CAG-Q	46	0.75	CGG-R	27	0.95
AUU-I	104	1.18	ACU-T	113	1.41	AAU-N	95	1.02	AGU-S	33	0.53
AUC-I	72	0.82	ACC-T	85	1.06	AAC-N	91	0.98	AGC-S	81	1.29
AUA-M	56	1.11	ACA-T	88	1.1	AAA-K	74	1.36	AGA-*	27	0.51
AUG-M	45	0.89	ACG-T	35	0.44	AAG-K	35	0.64	AGG-*	57	1.09
GUU-V	28	0.88	GCU-A	51	1.24	GAU-D	34	0.87	GGU-G	33	1.09
GUC-V	31	0.98	GCC-A	64	1.56	GAC-D	44	1.13	GGC-G	33	1.09
GUA-V	43	1.35	GCA-A	39	0.95	GAA-E	52	1.35	GGA-G	36	1.19
GUG-V	25	0.79	GCG-A	10	0.24	GAG-E	25	0.65	GGG-G	19	0.63
Cephalopholis miniata											
UUU-F	73	0.9	UCU-S	95	1.49	UAU-Y	71	0.93	UGU-C	21	0.64
UUC-F	89	1.1	UCC-S	69	1.08	UAC-Y	81	1.07	UGC-C	45	1.36
UUA-L	74	0.95	UCA-S	60	0.94	UAA-*	65	1.25	UGA-W	55	1.24
UUG-L	49	0.63	UCG-S	39	0.61	UAG-*	54	1.04	UGG-W	34	0.76
CUU-L	104	1.34	CCU-P	166	1.66	CAU-H	94	1.06	CGU-R	26	0.89
CUC-L	71	0.91	CCC-P	116	1.16	CAC-H	84	0.94	CGC-R	39	1.33
CUA-L	106	1.36	CCA-P	91	0.91	CAA-Q	81	1.31	CGA-R	26	0.89
CUG-L	62	0.8	CCG-P	26	0.26	CAG-Q	43	0.69	CGG-R	26	0.89
AUU-I	105	1.14	ACU-T	119	1.5	AAU-N	91	1.03	AGU-S	36	0.57
AUC-I	79	0.86	ACC-T	75	0.94	AAC-N	86	0.97	AGC-S	83	1.3
AUA-M	53	1.02	ACA-T	91	1.14	AAA-K	75	1.38	AGA-*	29	0.56
AUG-M	51	0.98	ACG-T	33	0.42	AAG-K	34	0.62	AGG-*	60	1.15
GUU-V	34	1.07	GCU-A	53	1.28	GAU-D	34	0.86	GGU-G	34	1.12
GUC-V	22	0.69	GCC-A	66	1.59	GAC-D	45	1.14	GGC-G	31	1.02
GUA-V	48	1.51	GCA-A	38	0.92	GAA-E	53	1.38	GGA-G	33	1.09
GUG-V	23	0.72	GCG-A	9	0.22	GAG-E	24	0.62	GGG-G	23	0.76
Cephalopholis sexmaculata											

UUU-F	80	0.99	UCU-S	87	1.42	UAU-Y	79	0.99	UGU-C	26	0.71
UUC-F	82	1.01	UCC-S	70	1.14	UAC-Y	81	1.01	UGC-C	47	1.29
UUA-L	84	1.08	UCA-S	59	0.96	UAA-*	72	1.3	UGA-W	47	1.16
UUG-L	42	0.54	UCG-S	37	0.6	UAG-*	52	0.94	UGG-W	34	0.84
CUU-L	106	1.36	CCU-P	164	1.64	CAU-H	92	1.05	CGU-R	27	0.99
CUC-L	86	1.1	CCC-P	125	1.25	CAC-H	84	0.95	CGC-R	36	1.32
CUA-L	88	1.13	CCA-P	84	0.84	CAA-Q	85	1.25	CGA-R	26	0.95
CUG-L	62	0.79	CCG-P	28	0.28	CAG-Q	51	0.75	CGG-R	20	0.73
AUU-I	103	1.15	ACU-T	107	1.4	AAU-N	97	1.07	AGU-S	34	0.55
AUC-I	76	0.85	ACC-T	79	1.03	AAC-N	84	0.93	AGC-S	81	1.32
AUA-M	56	1.04	ACA-T	86	1.12	AAA-K	72	1.4	AGA-*	34	0.62
AUG-M	52	0.96	ACG-T	34	0.44	AAG-K	31	0.6	AGG-*	63	1.14
GUU-V	38	1.19	GCU-A	55	1.28	GAU-D	29	0.78	GGU-G	28	0.89
GUC-V	23	0.72	GCC-A	66	1.53	GAC-D	45	1.22	GGC-G	41	1.3
GUA-V	48	1.5	GCA-A	41	0.95	GAA-E	52	1.42	GGA-G	30	0.95
GUG-V	19	0.59	GCG-A	10	0.23	GAG-E	21	0.58	GGG-G	27	0.86
Cephalopholis sonnerati											
UUU-F	86	1.04	UCU-S	90	1.49	UAU-Y	69	0.94	UGU-C	20	0.63
UUC-F	79	0.96	UCC-S	75	1.24	UAC-Y	78	1.06	UGC-C	43	1.37
UUA-L	86	1.07	UCA-S	58	0.96	UAA-*	71	1.26	UGA-W	51	1.2
UUG-L	47	0.59	UCG-S	25	0.41	UAG-*	58	1.03	UGG-W	34	0.8
CUU-L	103	1.28	CCU-P	168	1.72	CAU-H	101	1.09	CGU-R	28	0.97
CUC-L	84	1.05	CCC-P	111	1.14	CAC-H	85	0.91	CGC-R	42	1.45
CUA-L	98	1.22	CCA-P	85	0.87	CAA-Q	86	1.31	CGA-R	25	0.86
CUG-L	63	0.79	CCG-P	26	0.27	CAG-Q	45	0.69	CGG-R	21	0.72
AUU-I	112	1.18	ACU-T	104	1.39	AAU-N	92	0.99	AGU-S	34	0.56
AUC-I	78	0.82	ACC-T	78	1.04	AAC-N	93	1.01	AGC-S	80	1.33
AUA-M	55	0.98	ACA-T	80	1.07	AAA-K	76	1.42	AGA-*	33	0.59
AUG-M	57	1.02	ACG-T	37	0.49	AAG-K	31	0.58	AGG-*	63	1.12
GUU-V	31	0.99	GCU-A	53	1.25	GAU-D	35	0.88	GGU-G	38	1.33

GUC-V	24	0.77	GCC-A	66	1.56	GAC-D	45	1.13	GGC-G	29	1.02
GUA-V	52	1.66	GCA-A	40	0.95	GAA-E	52	1.42	GGA-G	32	1.12
GUG-V	18	0.58	GCG-A	10	0.24	GAG-E	21	0.58	GGG-G	15	0.53
Cephalopholis urodeta											
UUU-F	82	1.03	UCU-S	97	1.51	UAU-Y	73	0.94	UGU-C	27	0.74
UUC-F	77	0.97	UCC-S	78	1.22	UAC-Y	83	1.06	UGC-C	46	1.26
UUA-L	77	0.98	UCA-S	64	1	UAA-*	73	1.32	UGA-W	49	1.21
UUG-L	42	0.54	UCG-S	31	0.48	UAG-*	56	1.01	UGG-W	32	0.79
CUU-L	104	1.33	CCU-P	156	1.6	CAU-H	99	1.11	CGU-R	21	0.77
CUC-L	82	1.05	CCC-P	118	1.21	CAC-H	79	0.89	CGC-R	40	1.47
CUA-L	103	1.31	CCA-P	85	0.87	CAA-Q	85	1.29	CGA-R	29	1.06
CUG-L	62	0.79	CCG-P	31	0.32	CAG-Q	47	0.71	CGG-R	19	0.7
AUU-I	107	1.17	ACU-T	110	1.45	AAU-N	90	0.97	AGU-S	34	0.53
AUC-I	76	0.83	ACC-T	76	1	AAC-N	95	1.03	AGC-S	81	1.26
AUA-M	54	1.01	ACA-T	77	1.01	AAA-K	71	1.35	AGA-*	34	0.62
AUG-M	53	0.99	ACG-T	41	0.54	AAG-K	34	0.65	AGG-*	58	1.05
GUU-V	23	0.75	GCU-A	68	1.56	GAU-D	39	1.04	GGU-G	36	1.23
GUC-V	30	0.98	GCC-A	57	1.31	GAC-D	36	0.96	GGC-G	33	1.13
GUA-V	51	1.66	GCA-A	44	1.01	GAA-E	54	1.38	GGA-G	24	0.82
GUG-V	19	0.62	GCG-A	5	0.11	GAG-E	24	0.62	GGG-G	24	0.82
Cephalopholis spiloparaea											
UUU-F	74	0.91	UCU-S	93	1.46	UAU-Y	71	0.94	UGU-C	20	0.65
UUC-F	88	1.09	UCC-S	70	1.1	UAC-Y	80	1.06	UGC-C	42	1.35
UUA-L	74	0.95	UCA-S	61	0.96	UAA-*	66	1.27	UGA-W	54	1.21
UUG-L	49	0.63	UCG-S	38	0.6	UAG-*	52	1	UGG-W	35	0.79
CUU-L	104	1.34	CCU-P	165	1.66	CAU-H	95	1.06	CGU-R	28	0.9
CUC-L	72	0.93	CCC-P	117	1.18	CAC-H	84	0.94	CGC-R	43	1.39
CUA-L	107	1.37	CCA-P	89	0.89	CAA-Q	80	1.3	CGA-R	26	0.84
CUG-L	61	0.78	CCG-P	27	0.27	CAG-Q	43	0.7	CGG-R	27	0.87
AUU-I	107	1.16	ACU-T	118	1.48	AAU-N	93	1.04	AGU-S	36	0.57

AUC-I	77	0.84	ACC-T	77	0.97	AAC-N	86	0.96	AGC-S	83	1.31
AUA-M	53	1.02	ACA-T	90	1.13	AAA-K	75	1.39	AGA-*	29	0.56
AUG-M	51	0.98	ACG-T	33	0.42	AAG-K	33	0.61	AGG-*	61	1.17
GUU-V	34	1.07	GCU-A	53	1.27	GAU-D	35	0.89	GGU-G	33	1.11
GUC-V	22	0.69	GCC-A	66	1.58	GAC-D	44	1.11	GGC-G	30	1.01
GUA-V	47	1.48	GCA-A	40	0.96	GAA-E	54	1.42	GGA-G	32	1.08
GUG-V	24	0.76	GCG-A	8	0.19	GAG-E	22	0.58	GGG-G	24	0.81

Table S8. Detailed comparison of anticodons found in the transfer RNA genes within the nine *Cephalopholis* mitogenomes. The duplication of tRNA-Asp (D) in *C. argus* is highlighted in gray colour.

Genes	*C. taeniops*	*C. argus*	*C. boenak*	*C. leopardus*	*C. miniata*	*C. sexmaculata*	*C. sonnerati*	*C. urodeta*	*C. spiloparaea*
tRNA-Phe (F)	TTC	TTC	TTC	TTC	TTC	TTC	TTC	TTC	TTC
tRNA-Val (V)	GTA	GTA	GTA	GTA	GTA	GTA	GTA	GTA	GTA
tRNA-Leu (L2)	TTA	TTA	TTA	TTA	TTA	TTA	TTA	TTA	TTA
tRNA-Ile (I)	ATC	ATC	ATC	ATC	ATC	ATC	ATC	ATC	ATC
tRNA-Gln (Q)	CAA	CAA	CAA	CAA	CAA	CAA	CAA	CAA	CAA
tRNA-Met (M)	ATG	ATG	ATG	ATG	ATG	ATG	ATG	ATG	ATG
tRNA-Trp (W)	TGA	TGA	TGA	TGA	TGA	TGA	TGA	TGA	TGA
tRNA-Ala (A)	GCA	GCA	GCA	GCA	GCA	GCA	GCA	GCA	GCA
tRNA-Asn (N)	AAC	AAC	AAC	AAC	AAC	AAC	AAC	AAC	AAC
tRNA-Cys (C)	TGC	TGC	TGC	TGC	TGC	TGC	TGC	TGC	TGC
tRNA-Tyr (Y)	TAC	TAC	TAC	TAC	TAC	TAC	TAC	TAC	TAC
tRNA-Ser (S2)	TCA	TCA	TCA	TCA	TCA	TCA	TCA	TCA	TCA
tRNA-Asp (D)	GAC	GAC	GAC	GAC	GAC	GAC	GAC	GAC	GAC
tRNA-Lys (K)	AAA	AAA	AAA	AAA	AAA	AAA	AAA	AAA	AAA
tRNA-Gly (G)	GGA	GGA	GGA	GGA	GGA	GGA	GGA	GGA	GGA
tRNA-Arg (R)	CGA	CGA	CGA	CGA	CGA	CGA	CGA	CGA	CGA
tRNA-His (H)	CAC	CAC	CAC	CAC	CAC	CAC	CAC	CAC	CAC
tRNA-Ser (S1)	AGC	AGC	AGC	---	---	---	---	---	---
tRNA-Leu (L1)	CTA	CTA	CTA	CTA	CTA	CTA	CTA	CTA	CTA
tRNA-Glu (E)	GAA	GAA	GAA	GAA	GAA	GAA	GAA	GAA	GAA
tRNA-Thr (T)	ACA	ACA	ACA	ACA	ACA	ACA	ACA	ACA	ACA
tRNA-Pro (P)	CCA	CCA	CCA	CCA	CCA	CCA	CCA	CCA	CCA
tRNA-Asp (D)		GAC							

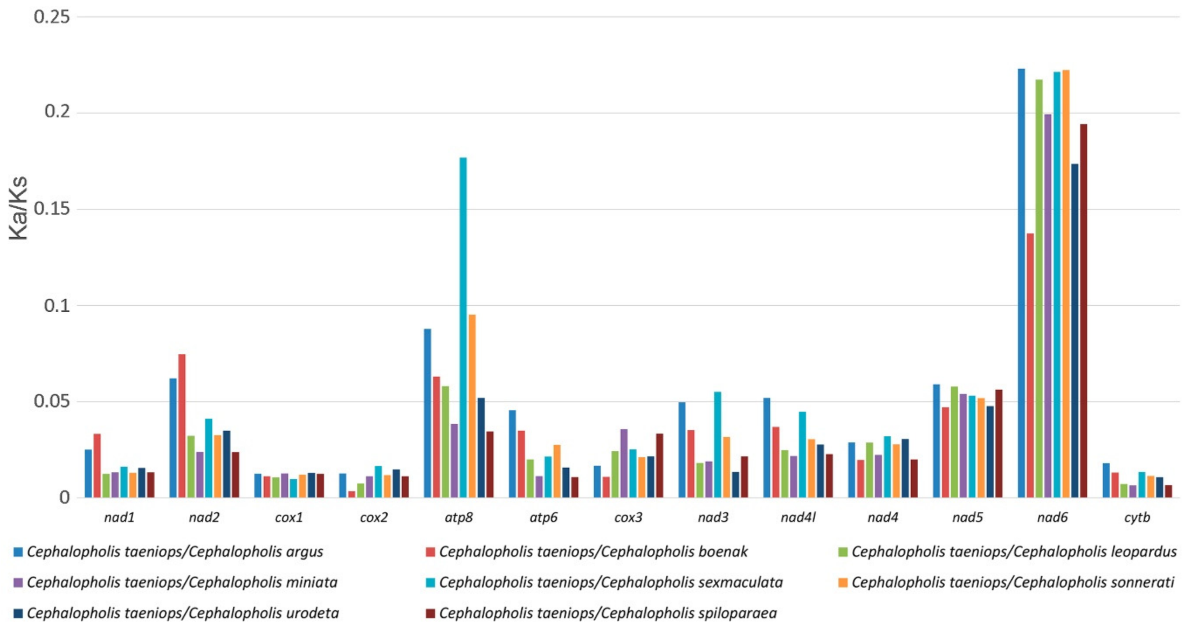

Figure S1. Evolutionary rates (Ka/Ks) of individuals PCGs of *C. taeniops* with other *Cephalopholis* species.

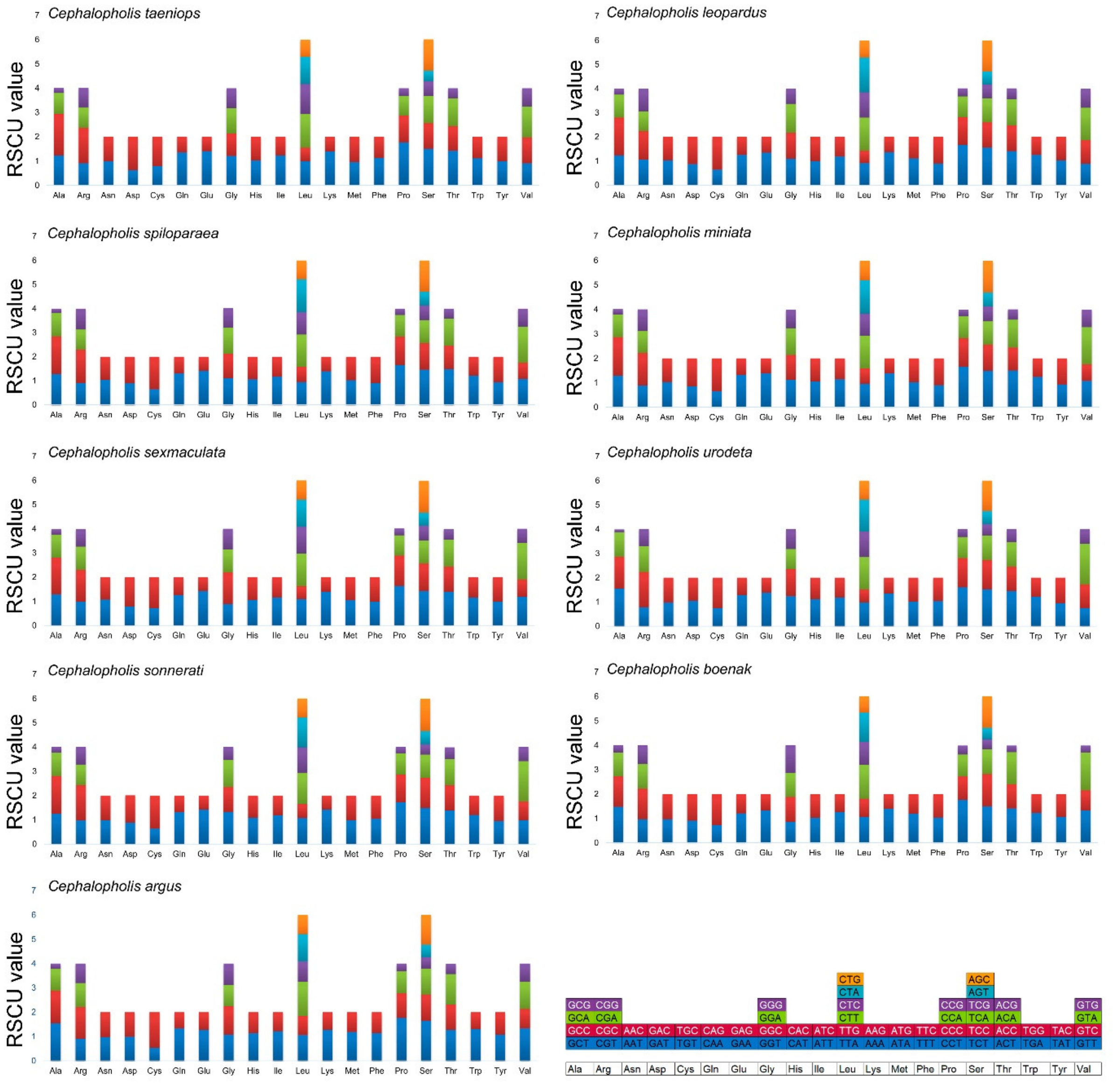

Figure S2. Comparative Relative synonymous codon usage (RSCU) in *Cephalopholis* species including *C. taeniops*. The cumulative RSCU values are represented on the y-axis while the codon families for each amino acid are represented on the x-axis.

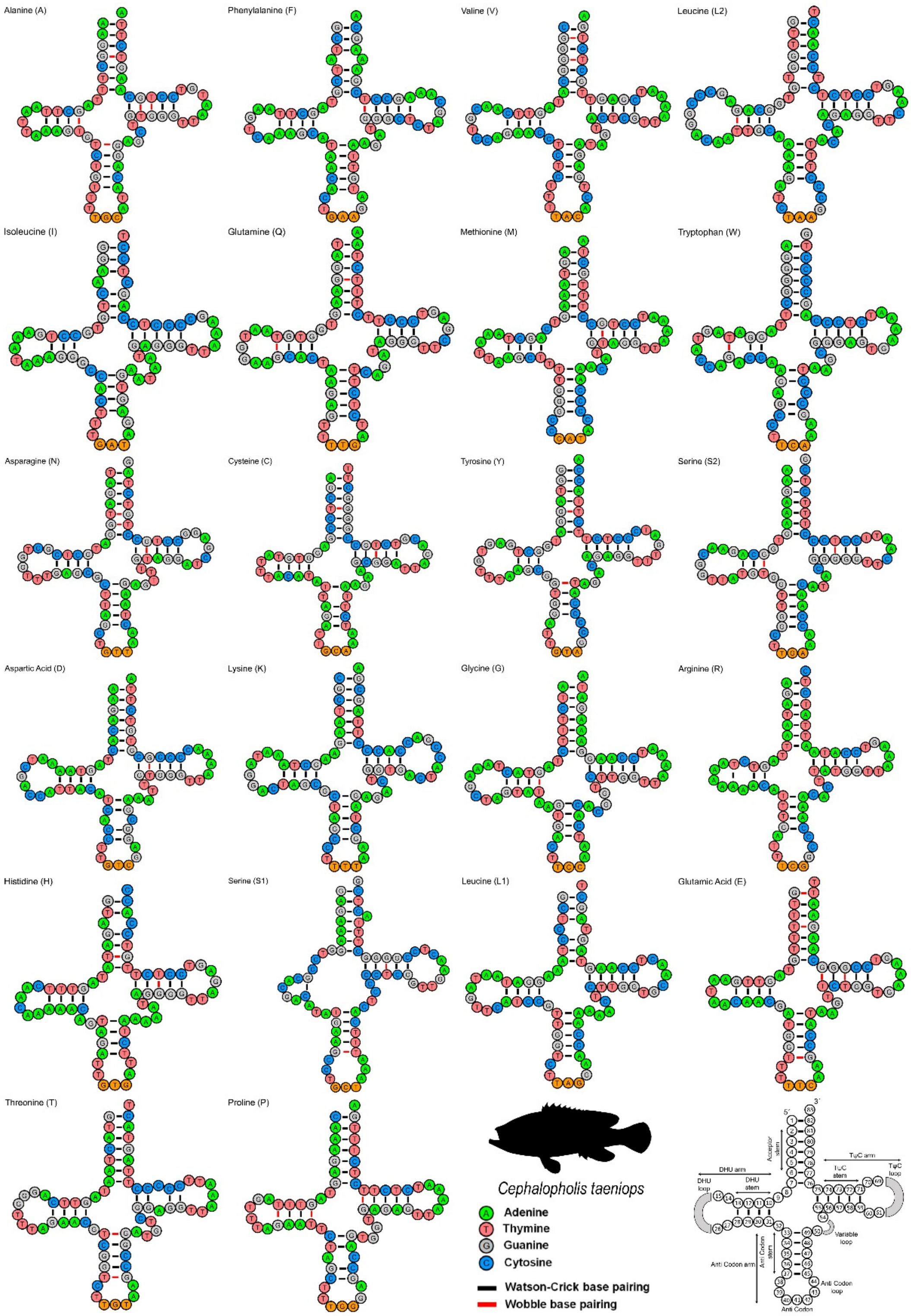

Figure S3. The secondary structures of 22 transfer RNAs (tRNAs) in the African Hind, *C. taeniops* mitogenome exhibit structural variations. These tRNAs are labelled with their complete names and single-letter amino acid codes following the IUPAC-IUB convention. The last structure provides information on the nucleotide positions and specifics of the tRNAs' stem-loop configuration.

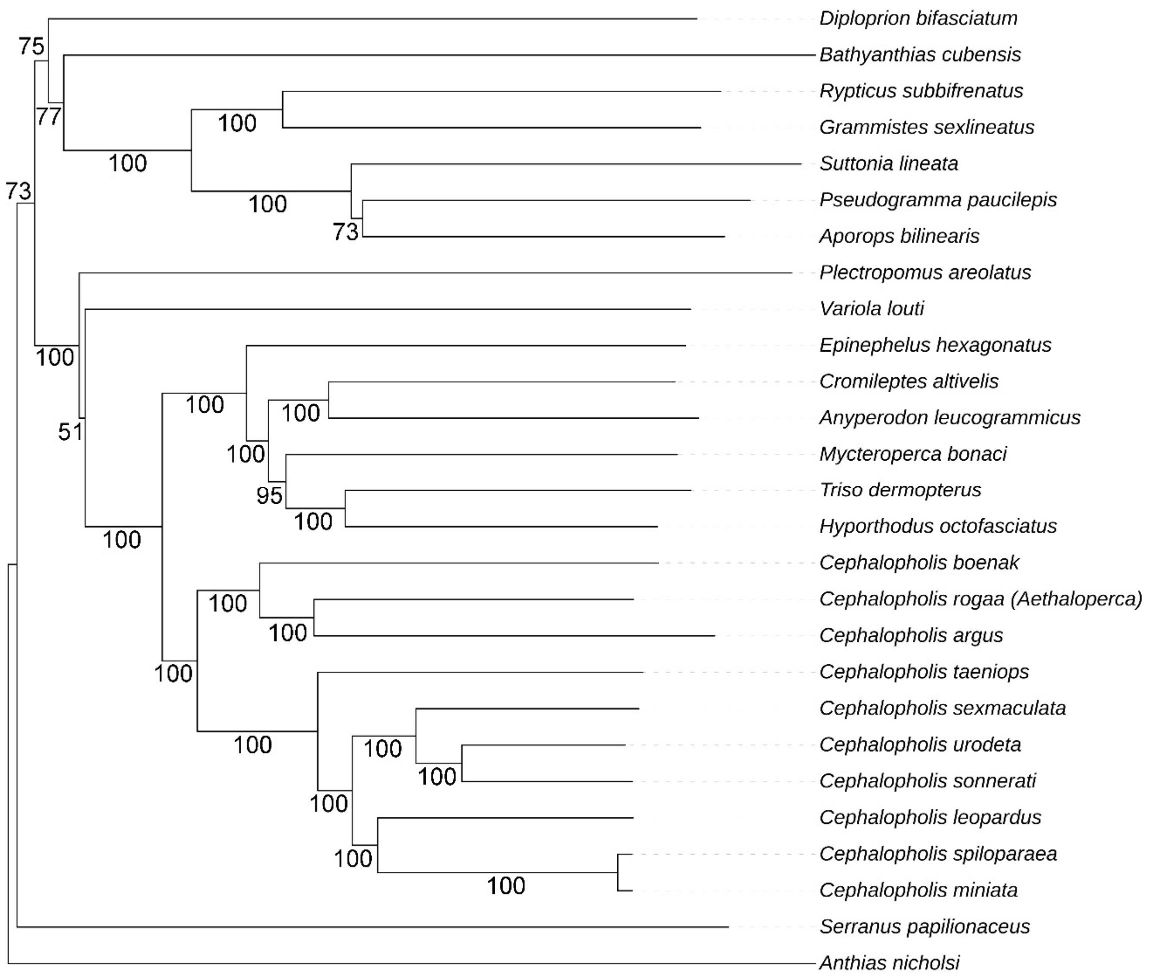

Figure S4. The neighbor-joining (NJ) phylogeny constructed by 13 concatenated PCGs clearly discriminate *C. taeniops* and other *Cephalopholis* congeners.

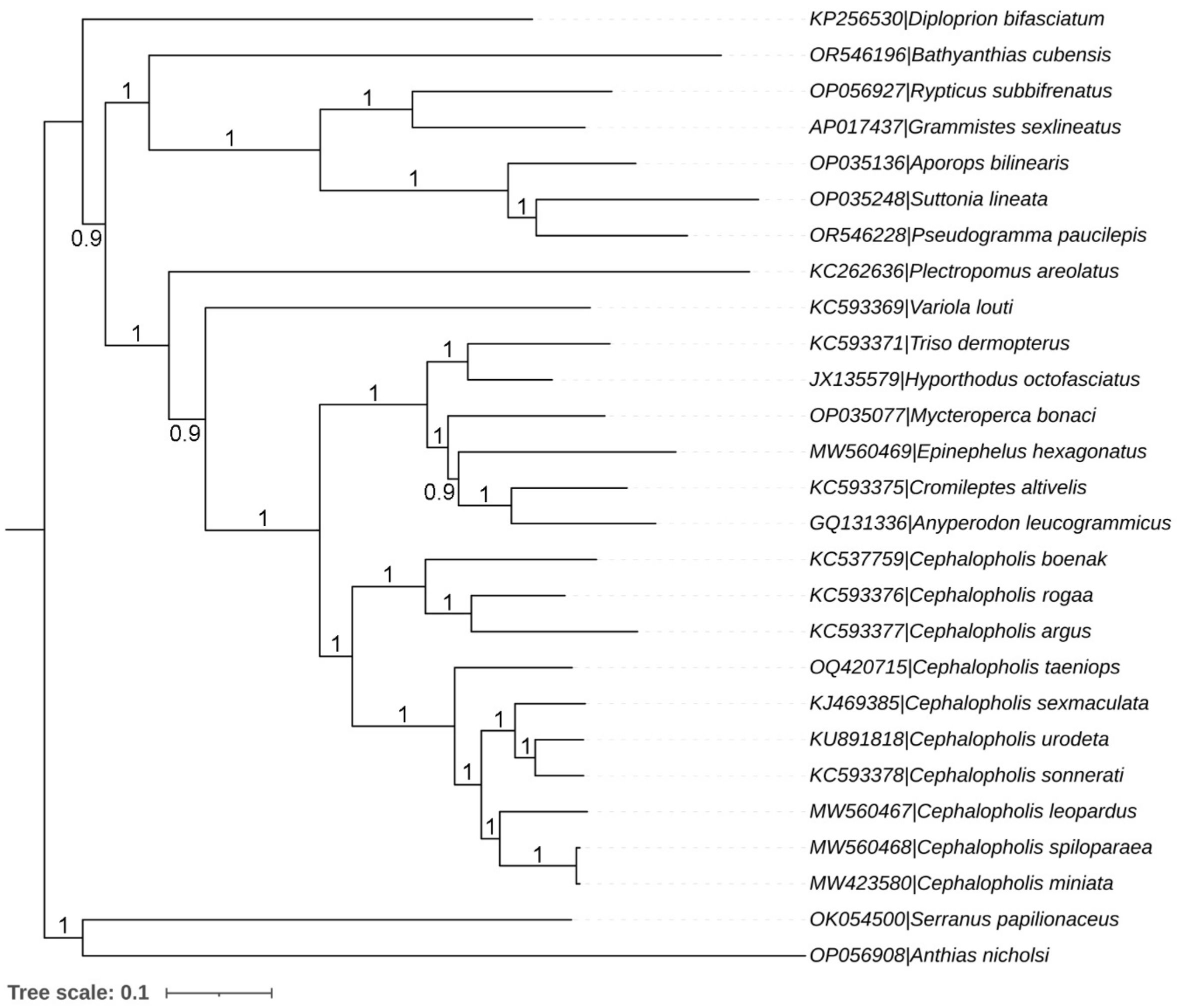

Figure S5. The Bayesian (BA) phylogeny constructed by 13 concatenated PCGs clearly discriminate *C. taeniops* and other *Cephalopholis* congeners.

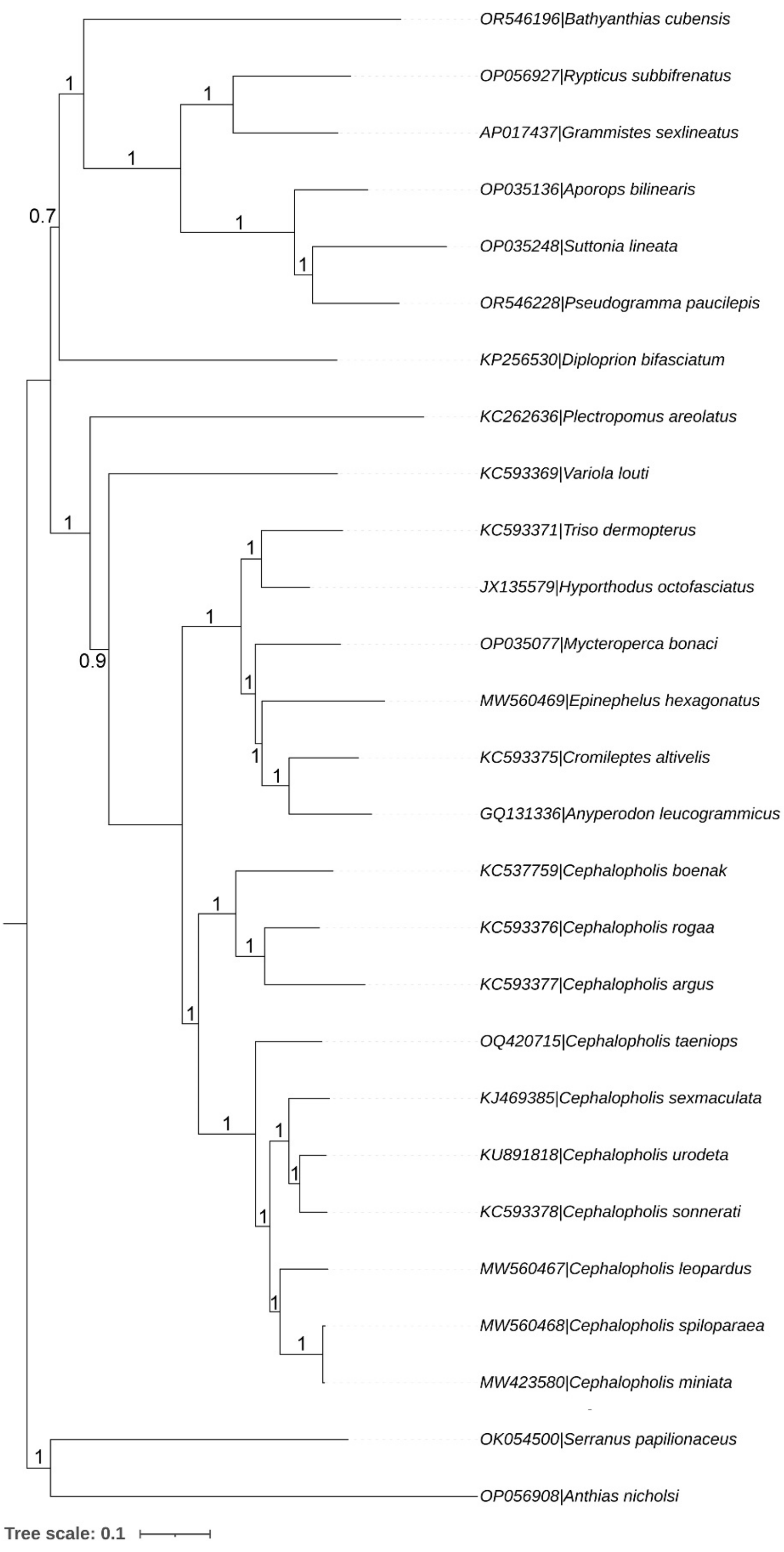

Figure S6. The Bayesian (BA) phylogeny constructed by 13 concatenated PCGs and two rRNAs clearly discriminate *C. taeniops* and other *Cephalopholis* congeners.
